# Molecularly Imprinted Viral Protein Integrated Zn–Cu–In–Se–P Quantum Dots Superlattice for Quantitative Ratiometric Electrochemical Detection of SARS-CoV-2 Spike Protein in Saliva

**DOI:** 10.1021/acsanm.4c02882

**Published:** 2024-07-24

**Authors:** Kayode Omotayo Adeniyi, Kayode Oyinlola, Ojodomo J. Achadu, Herve Menard, Federico Grillo, Zhugen Yang, Oluwasesan Adegoke

**Affiliations:** † Leverhulme Research Centre for Forensic Science, School of Science & Engineering, University of Dundee, Dundee DD1 4GH, U.K.; ‡ School of Health and Life Sciences, and National Horizon Centre, Teesside University, Middlesbrough TS1 3BA, U.K.; § School of Water, Energy and Environment, Cranfield University, Cranfield MK43 0AL, U.K.; ∥ School of Chemistry, University of St Andrews, St Andrews KY16 9ST, U.K.

**Keywords:** Zn–Cu–In–P–Se quantum dots, protein imprinted polymer, SARS-CoV-2 detection, ratiometric sensor, quantum dot superlattice

## Abstract

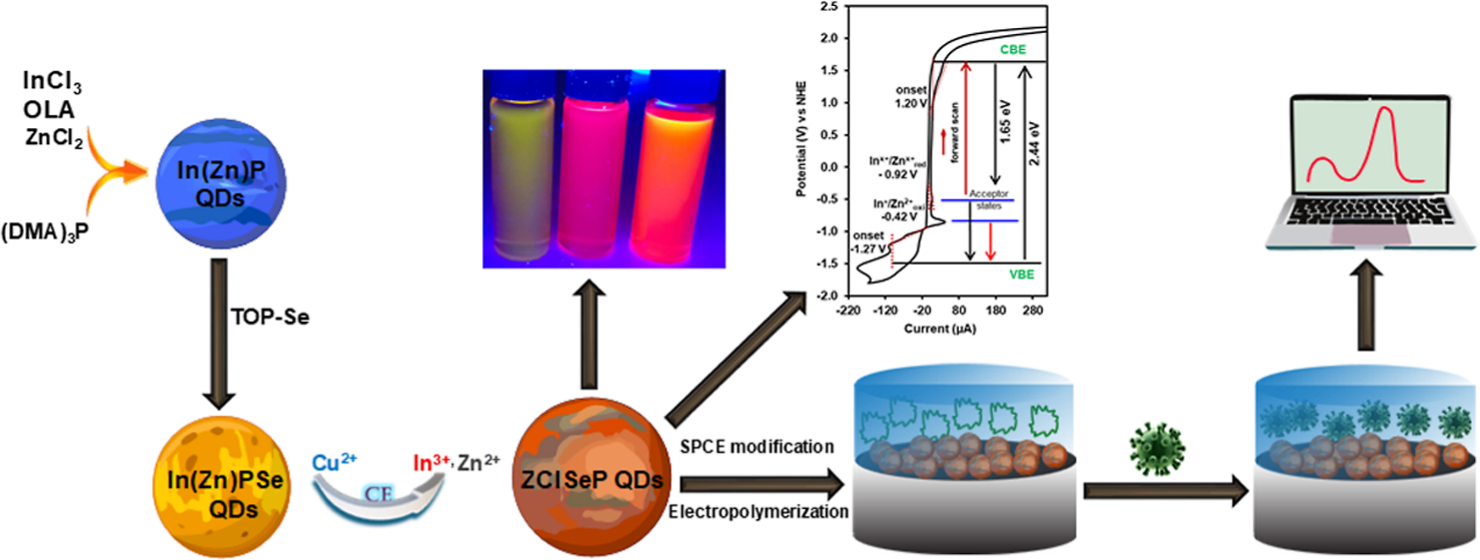

Solution-processable colloidal quantum dots (QDs) are promising materials for the development of rapid and low-cost, next-generation quantum-sensing diagnostic systems. In this study, we report on the synthesis of multinary Zn–Cu–In–Se–P (ZCISeP) QDs and the application of the QDs-modified electrode (QDs/SPCE) as a solid superlattice transducer interface for the ratiometric electrochemical detection of the SARS-CoV-2-S1 protein in saliva. The ZCISeP QDs were synthesized through the formation of In(Zn)PSe QDs from InP QDs, followed by the incorporation of Cu cations into the crystal lattice via cation exchange processes. A viral-protein-imprinted polymer film was deposited onto the QDs/SPCE for the specific binding of SARS-CoV-2. Molecular imprinting of the virus protein was achieved using a surface imprinting electropolymerization strategy to create the MIP@QDs/SPCE nanosensor. Characterization through spectroscopic, microscopic, and electrochemical techniques confirmed the structural properties and electronic-band state of the ZCISeP QDs. Cyclic voltammetry studies of the QDs/SPCE superlattice confirmed efficient electron transport properties and revealed an intraband gap energy state with redox peaks attributed to the Cu^1+/2+^ defects. Binding of SARS-CoV-2-S1 to the MIP@QDs/SPCE cavities induced a gating effect that modulated the Fe(CN)_6_^3–/4–^ and Cu^1+/2+^ redox processes at the nanosensor interface, producing dual off/on ratiometric electrical current signals. Under optimal assay conditions, the nanosensor exhibited a wide linear detection range (0.001–100 pg/mL) and a low detection limit (0.34 pg/mL, 4.6 fM) for quantitative detection of SARS-CoV-2-S1 in saliva. The MIP@QDs/SPCE nanosensor demonstrated excellent selectivity against nonspecific protein targets, and the integration with a smartphone-based potentiostat confirmed the potential for point-of-care applications.

## 1 Introduction

The increasing frequency and severity of global health crises caused by pathogenic viruses, such as the 2019 SARS-CoV-2 pandemic, underscore the need for next-generation nanosensors designed for efficient virus detection. These sensors should meet the ASSURED criteria, which encompasses being affordable, sensitive, selective, user-friendly, rapid and robust, equipment-free, and deliverable to end-users for self-testing quantification.^1^ Meeting these criteria is crucial for implementing effective public health safety measures aimed at early diagnosis, surveillance, and control of infectious viral diseases transmission.^1,2^ Traditional virus detection methods, such as real-time reverse transcription polymerase chain reaction and enzyme-linked immunosorbent assay, offer high sensitivity and specificity, but do not meet the ASSURED criteria due to their high costs, bulky instrumentation, the need for technical experts, expensive reagents, and a long processing times.^3^ Various low-cost and rapid detection methods, such as lateral flow antibodies and antigen-based colorimetric^4−6^ and fluorescence-based systems,^7−9^ have been developed. However, these methods often suffer from low sensitivity and require bulky readout equipment for qualitative measurement.

Electrochemical affinity biosensors utilizing RNA/DNA probes and antibody/antigen-mediated target binding, offer a unique opportunity for rapid and accurate point-of-care diagnostic sensing systems using inexpensive portable or hand-held potentiostat.^10^ Nevertheless, their clinical applications are limited by low sensitivity and a high rate of false positives and false negatives due to the degradation of antibodies and RNA probes over time.^11^ Additionally, the production of DNA probes and antibodies is expensive and exhibits a poor batch-to-batch variation. An emerging alternative to overcome these limitations is the development of an antibody-like artificial receptor through molecularly imprinted polymer (MIP) technology.^12^ MIPs are synthesized by copolymerizing a monomer, cross-linker, and template (target analyte), resulting in the formation of molecularly imprinted cavities that are complementary, in shape, size, and chemical environment to the target analyte upon template removal.^13^ These MIP cavities mimic the lock and key activities of the paratopes on an antibody, making them viable recognition sites with high specificity for a target. However, imprinting the entire virion remains challenging due to their large and complex polypeptide structure and denaturing of their native 3D conformation during the imprinting process. Additionally, the large molecular size of the whole virion makes their extraction from the polymer matrix difficult, leading to a low yield of specific binding sites.^14^ Therefore, imprinting viral surface protein subunits has been pursued for sensor development.

SARS-CoV-2 has four major structural proteins: the spike (S), envelop (E), and membrane (M) are surface proteins, while the nucleocapsid (N) proteins are embedded inside the virion.^15^ The S protein has an S1 subunit with a receptor binding domain (RBD) that plays a crucial role in the virus binding to the host entry receptor angiotensin-converting enzyme 2, and the cell membrane. Several electrochemical sensors based on surface imprinted SARS-CoV-2 nucleoprotein,^16,17^ SARS-CoV-2 RBD,^18−21^ and SARS-CoV-2 S1 spike protein,^20^ have been developed for SARS-CoV-2 detection. However, these sensors typically rely on absolute values of a single electrochemical signal, leading to less reliable results and poor reproducibility. Ratiometric electrochemical sensors, which possess dual electrochemical signals, offer improved accuracy and reliability by inherently correcting for nonspecific interferences. Despite these advantages, no ratiometric MIP-based electrochemical nanosensors for the detection of SARS-CoV-2 have been reported.

Nanomaterial-modified transducer interfaces enhance biomolecule enrichment due to their high surface area,^22^ leading to highly sensitive detection.^23−25^ Herein, we hypothesize that integrating semiconductor quantum dots (QDs) superlattices with MIPs can offer unique transducer functionality for the development of highly sensitive and reliable ratiometric electrochemical nanosensors for SARS-CoV-2. QDs are zero-dimensional metal chalcogenides with particle sizes smaller than their excitonic Bohr radius, exhibiting unique optical and electronic properties due to quantum mechanical effects. In these QDs, electrons, and excitons are confined in all three spatial dimensions, resulting in quantized energy states and discrete band gaps in their density of states, unlike the continuous band of states in bulk materials.^26,27^ Owing to these unique “atom-like” properties and their solution processability, QDs have emerged as promising materials for the development of next-generation molecular diagnostics and quantum sensing systems. Among various quantum materials, multinary QDs incorporating group I–III–VI elements, such as Cu-GA-S, In–Ga–As–Sb,^28^ Zn–Ag–In–Ga–S,^29,30^ Zn–Ag–Sn–S,^31,32^ Zn–Cu–Ga–Se–S^33^ and Cu–Zn–Sn–S,^34^ have gained interest due to their low toxicity compared to conventional toxic cadmium (Cd) and lead (Pb)-based QDs.^35^ The ability to precisely adjust the electronic state and energy band gap of multinary QDs by altering their elemental composition ratio allows effective turnability of their properties to achieve specific functionalities.^36^ However, synthesizing high-quality phosphide-based multinary QDs via traditional heat-up and hot-injection methods has proven challenging due to the disparate reactivity of precursors,^37^ resulting in complex equilibria between anionic and cationic species and the formation of undesired byproducts.^38^ Phosphide-based QDs offer superior carrier mobility due to more effective quantum confinement, lower effective masses, and fewer impurity states compared with metal-oxide QDs. These characteristics make phosphide-based QDs more suitable for high-performance electronic applications. Consequently, new strategies for synthesizing high-quality multinary QDs are essential. When QDs are deposited onto a solid substrate, they assemble into closely packed quasi-2D crystalline superlattice solid film.^39−41^ This superlattice exhibits high electronic mobility and metallic properties due to the formation of interconnected miniband electronic states through a linear combination of subatomic orbitals.^42−44^ Due to these conductive characteristics and their high electroactive surface area, QDs-superlattice offers promising prospects as a functional transducer interface for the development of next-generation field-effect transistors and electrochemical nanosensors for the diagnosis and prognosis of infectious diseases.^45−48^

In this study, we report on the synthesis of new multinary Zn–Cu–In–Se–P (ZCISeP) QDs via a sequential partial cation exchange (CE) process starting from binary In(Zn)P QDs. We have demonstrated the development of an “on”/“off” dual signal ratiometric MIP-based electrochemical nanosensor for sensitive and reliable detection of SARS-CoV-2 S1 spike protein in human saliva employing ZCISeP QDs superlattice-modified screen-printed electrode for the first time. The QDs superlattice was functionalized with molecularly imprinted viral protein binding cavities to confer specificity for the SARS-CoV-2 spike S1 protein. The analytical performance of the nanosensor was evaluated, and the mechanism of dual electrochemical signals has been discussed. Integration of the sensor chip with a smartphone-based potentiostat resulted in a simple hand-held system capable of personalized SARS-CoV-2 testing, enabling timely monitoring and management of viral infection. This study represents a significant advancement in electrochemical sensing technology for virus detection, offering enhanced sensitivity and reliability in future clinical applications.

## 2 Experimental Section

### 2.1 Reagents and Materials

SARS-CoV-2 spike-S1-His (CoV-2_S1) protein generated by fusing the SARS-CoV-2 spike-S1[V16-R685] to a C-terminal polyhistidine sequence was purchased from InvivoGen (UK). The CoV_S1 viral sequence is from the original Wuhan-Hu-1 isolate and has the D614G mutation. SARS-CoV-2 envelope (COV-E) protein was purchased from the Medical Research Council (MRC) Protein Phosphorylation and Ubiquitylation Unit, University of Dundee. Recombinant influenza A hemagglutinin protein, recombinant dengue virus 1 NS1 protein, recombinant BK polyomavirus, strain AS, major capsid VP1 protein, and recombinant human IgG1 protein were purchased from Abcam. All the proteins have been validated using ELISA. Indium chloride (InCl_3_, 98%), copper(II) chloride dihydrate (CuCl_2_·2H_2_O), *cis*-9-octadecenoic acid sodium salt (sodium oleate, 99%), zinc chloride (ZnCl_2_, 98%), oleic acid (OA, 90%), oleylamine (OAm, 80%), trioctylphosphine (TOP, 97%), tris dimethylaminophosphine [(DMA)_3_P, 97%], selenium (Se, 99.9%), potassium acetate (NaAc), potassium ferricyanide (K_3_[Fe(CN)_6_], potassium ferrocyanide (K_4_[Fe(CN)_6_]), potassium chloride (KCl), *ortho*-phenylenediamine (OPD, 99.5%) were purchased from Merck. Absolute ethanol (99.5%), acetone, and chloroform were purchased from Merck (United Kingdom). Disposable screen-printed carbon electrodes (SPCEs) consisting of a printed carbon ink working electrode (*d* = 4 mm), silver ink printed pseudoreference, and gold carbon ink printed counter electrode deposited on a ceramic substrate were purchased from DropSens (model 220AT). Human saliva samples were collected following ethical procedures.

### 2.2 Apparatus and Instruments

Details regarding equipment are provided in the Supporting Information.

### 2.3 Preparation of Metal Precursors

#### 2.3.1 Selenium Precusor

Trioctylphosphine selenide precursor was used and prepared by dissolving selenium powder (0.12 g, 1.52 mmol) in TOP (5 mL) and sonicating on a water bath at 50 °C for complete dissolution. The solution was then stirred and maintained at that temperature before use.

#### 2.3.2 Copper-Oleate Precursor

The copper oleate (Cu-oleate) was prepared following a previously reported method for iron oleate,^49^ but with some modification. The detailed experimental procedures are presented in the Supporting Information.

### 2.4 Synthesis of In(Zn)PSe QDs

In a typical synthesis, 0.65 g (3 mmol) of InCl_3_, 0.41 g (3 mmol) of ZnCl_2,_ and 20 mL of oleylamine (OLA) were introduced into a three-neck round-bottom flask. The mixture was degassed under a slow stream of a N_2_ gas flow at 50 °C for 30 min. Subsequently, the temperature was raised to 180 °C, and 2.18 mL of tris(dimethylamino)phosphine (DMA)_3_P (12 mmol) was swiftly injected. The reaction was maintained at this temperature for 10 min to allow the nucleation and growth of the In(Zn)P QDs. Then, 2 mL of 2 mmol L^–1^ of TOP-Se precursor solution was injected, and the reaction was held at this temperature for 20 min to grow the In(Zn)PSe QDs. For characterization, an aliquot of the reaction mixture was cooled to room temperature, and the QDs were precipitated by adding ethanol followed by centrifugation at 4000 rpm for 5 min (min). The QDs were redispersed in chloroform and purified by repeated centrifugation with ethanol and acetone.

### 2.5 Synthesis of ZCISeP QDs

ZCISeP QDs were synthesized via a partial CE procedure using synthesized In(Zn)PSe QDs as a template. Briefly, the crude In(Zn)PSe QD reaction mixture, as described in the previous section, was cooled to 130 °C, and 2 mL of Cu-oleate precursor (1.0 mmol) solution was dropwise injected at a rate of 2 mL/h under rigorous stirring and slow stream of N_2_ gas. The temperature was then raised to 150 °C and maintained for 60 min. The reaction was quenched by cooling to room temperature and precipitated by the addition of an excess of ethanol. The crude ZCISeP QDs precipitate was redispersed in chloroform and purified via repeated centrifugation with a 1:9 (v/v) mixture of acetone/ethanol. The precipitates were collected by centrifugation at 4000 rpm for 5 min. The purified QDs were dried in a vacuum at room temperature and dispersed in *n*-hexane for storage.

### 2.6 QDs Surface Ligand Exchange

The long-chain hydrophobic organic surface ligands of the QDs were replaced with short thiol ligands (MPA) through a biphasic ligand exchange procedure.^50^ Briefly, 300 mg of ZCISeP were redispersed in 10 mL of hexane and added dropwise into 20 mL of methanol containing 3 mL of MPA (36.4 mmol), while stirring at room temperature and sonicated for 1 h. After the solution was allowed to stand for 30 min, the QDs migrated to the methanol phase, and the hexane layer was decanted. Chloroform was added to the methanol layer to purify the QDs by repeated dispersion and centrifugation to remove unreacted MPA and organic ligands, and then it was washed with acetone. ZCISeP QDs (10 mg) were dispersed in 1 mL of ethanol/H_2_O (1:1 v/v) to form a stable colloidal QDs solution.

### 2.7 Fabrication of QDs Superlattice-Modified SPCE (QDs/SPCE)

**Scheme 1:**
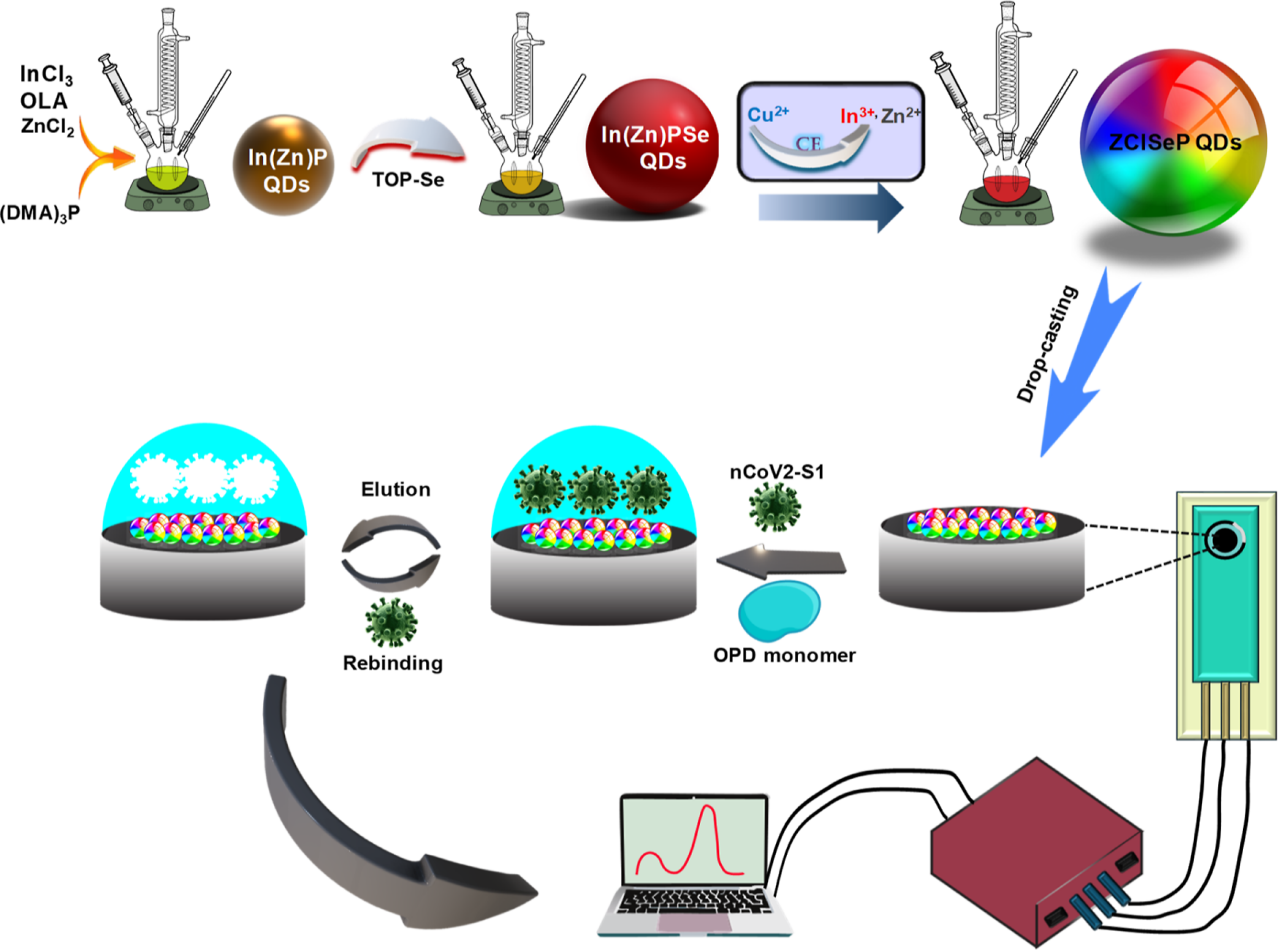
Diagrammatic Illustration Showing the Synthesis of ZCISeP QDs via Sequential Partial CE Procedure Starting from In(Zn)P QDs, and Subsequent Fabrication of the QDs/SPCE by Drop Casting, Electropolymerization of oPD and nCOV2-S1 Protein onto QDs/SPCE and Elution of nCOV2-S1 to form the MIP@QDs/SPCE Sensing Electrode

SPCE were subjected to electrochemical treatment by cycling in 0.5 M of H_2_SO_4_ solution between −1.2 and 1.2 V vs Ag|AgCl at 100 mV s^–1^. Following this, they were rinsed with Milli-Q water and dried in a stream of N_2_ gas. Next, 30 μL of the prepared QDs solution was drop-cast onto the circular working electrode area of the SPCE (Scheme 1). The electrodes were left to dry at room temperature to evaporate solvent and form a QD self-assembled film on the SPCE. Subsequently, the QDs modified SPCE was baked in an oven at 70 °C for 30 min. The ZCISeP QDs-modified SPCE is referred to as QDs/SPCE.

### 2.8 Sensor Surface Modification and nCoV_S1 Protein Imprinting

To demonstrate the applicability of ZCISeP QDs superlattice as a transducer interface for SARS-CoV-2 detection, the QDs/SPCE surface was functionalized with a MIP film containing nCoV_S1 protein-imprinted cavities as the recognition element via the surface imprinting electropolymerization method. Initially, an imprinting solution was prepared, comprising 10 mmol/L of OPD and 5.0 μg/mL of nCoV_S1 protein dissolved in acetate buffer (0.1 mol/L, pH 5.5), and incubated at 4 °C to ensure complete interactions between the nCoV_S1 protein and the OPD monomer. For the preparation of MIP@QDs/SPCE, 50 μL of the imprinting solution was dispensed onto the QDs/SPCE and incubated for 15 min to facilitate physisorption of the protein onto the QDs/SPCE. The mixture was then subjected to electrochemical polymerization via cyclic voltammetry. A potential ranging from −0.4 and 1.0 V was applied at a scan rate of 50 mV/s for 15 cycles of cyclic voltammogram (CV) scans to form a nCoV_S1-PoPD@QDs/SPCE. The nCoV_S1 template was eluted to create molecularly imprinted cavities specific to the nCoV_S1 protein by immersing the nCoV_S1-PoPD@QDs/SPCE in elution solution containing a mixture of 1:1 v/v ethanol and 0.25 mM NaOH, 135 mM NaCl, 1% SDS, 0.1% Tween 20 solution by stirring at 40 °C for 60 min. The electrode was further washed with Tris-HCl buffer (20 mM, pH 7.5, 0.1% Triton X-100) for 10 min under stirring to eliminate any remaining CoV_S1 template. The resulting fabricated electrode after template removal is denoted as MIP@QDs/SPCE (Scheme 1). As a control, a nonimprinted, NIP@QDs/SPCE, was prepared with the same molar concentrations of the OPD monomer but without including the CoV_S1 template using the same electropolymerization conditions.

## 3 Results and Discussion

### 3.1 Synthesis of ZCISeP QDs and Fabrication of QD@MIP/SPCE Sensor

Scheme 1 shows the schematic illustration of the synthesis of ZCISeP QDs with a nominal Zn/In/Cu molar ratio of 1:1:2 via sequential hot-injection organometallic-assisted partial CE process. The synthesis involves two steps. First, In(Zn)PSe QDs serving as the template for the synthesis of ZCISeP QDs, were synthesized by injecting the phosphine precursor, (DAM)_3_P, into a preheated cationic mixture of Zn^2+^ and In^3+^ with a nominal ratio of 1:1, using oleylamine as a coordinating solvent. Previous studies indicate that Zn^2+^ ions incorporate as substitutional dopants into the InP QDs crystal lattice, forming shorter Zn–P bonds compared to In–P bonds and simultaneously passivate surface defects and trap states^51,52^ and Zn^2+^ ions are more abundant on the surface of In(Zn)P QDs.^53^ Subsequently, a thin Se-rich layer is incorporated into the In(Zn)P QDs by rapidly injecting a TOP-Se precursor, resulting in the formation of an alloyed In(Zn)PSe. To avoid the generation of separate ZnSe QDs and In(Zn)PSe@ZnSe core–shell structure, the reaction temperature was maintained below 180 °C after the TOP-Se precursor injection. In the second step, the TOP-Cu precursor, prepared in octylamine was added dropwise into the pregrown In(Zn)PSe QDs crude solution at 150 °C. The Se layer acting as a soft base binds the Cu ions (a soft acid), facilitating Cu^2+^ adsorption onto the pregrown In(Zn)PSe QDs. The Cu^2+^ diffuses and incorporates into the In(Zn)PSe QDs lattice during which In^3+^ and Zn^2+^ cations are partially exchanged by Cu^+^ ions via substitutional CE process due to thermodynamic driving forces and similar ionic radius of Zn^2+^ (74 pm), Cu^2+^ (73 pm) and In^3+^ (80 pm). To prevent the generation of separate Cu_*x*_P or Cu_*x*_Se QDs, the Cu precursor was added dropwise and the temperature was lowered to 150 °C during the CE process. It would be important to mention that our proof-of-concept studies involving one-pot hot organometallic injection of the anionic species (P and Se precursors) into a heated mixture of Cu, In, Zn precursors resulted in the formation of agglomerated black precipitate immediately after the anion injections. This black precipitate was identified as a mixture of copper phosphide (Cu_*x*_P) and copper selenide (Cu_*x*_Se) nanoparticles (data not shown). The sequential partial CE procedure achieved the successful synthesis of ZCISeP QDs. The as-synthesized QDs were capped with long aliphatic hydrophobic oleylamine ligands. These long-chain surface ligands were substituted with short-chain thiol ligand (MPA) via biphasic ligand exchange transfer, which brings the surface exchanged QDs into a polar solvent (methanol). This ligand exchange is crucial for enhancing the conductivity and biocompatibility of the QDs/SPCE. The FTIR spectra of ZCISeP QDs before and after ligand exchange with MPA are shown in Figure S1a. The FTIR spectra of the as-synthesized ZCISeP QDs showed strong C–H stretching vibrations at 2922 and 2851 cm^–1^, attributed to the long aliphatic chain of the organic ligand.^54^ However, the peak intensity was significantly reduced after the ligand exchange process, confirming the replacement of the long aliphatic organic ligands of the ZCISeP QDs with short MPA ligands.

The MPA-capped ZCISeP QDs were cast onto the SPCE to form the QDs/SPCE. The CV of QD/SPCE in 10 mM oPD solution containing 5 μg/mL of nCoV2_S1 in Figure S1b showed irreversible anodic peaks (A) at −0.18, (B) at 0.26 and (C) at 0.54 V vs Ag|AgCl. These peaks correspond to the dimerization of oPD (A), oxidation of the dimer to its semioxidized state (B), and subsequent oxidation to polymerized 1,4-substituted benzenoid-quinoid structure (c).^55^ The decrease in the anodic peak current with an increasing number of CV scans confirmed the deposition of a nonconductive PoPD-nCoV2_S1 film onto the QD/SPCE without pinholes. The PoPD-nCov2_S1@QD/SPCE was treated with an elution buffer to remove the nCoV2_S1, leaving imprinted cavities that are complementary in size, shape, and chemical functionality to nCoV2_S1.^56,57^ These cavities act as specific recognition sites within the MIP interface for the affinitive binding of nCov2_S1 spike proteins, as shown in Scheme 1.

### 3.2 Characterization of ZCISeP QDs

**Figure 1:**
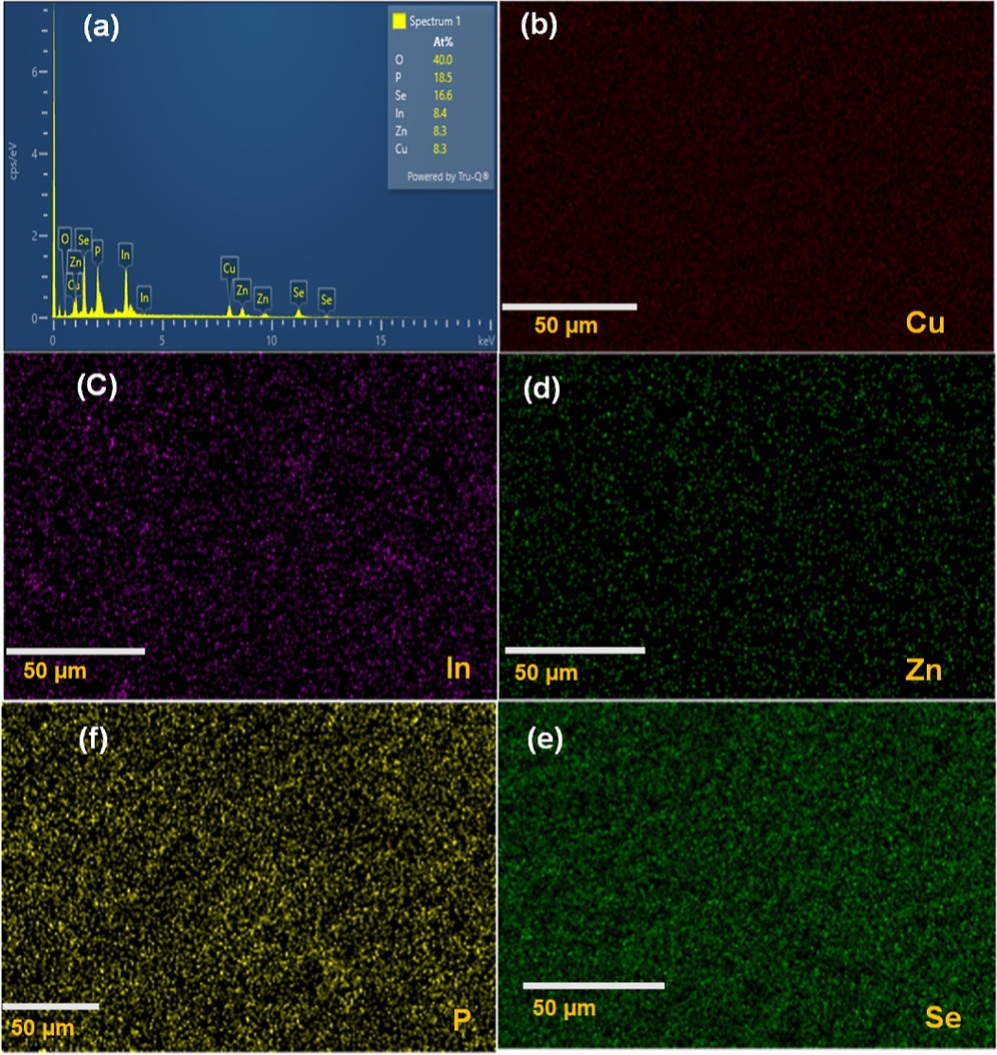
(a) SEM–EDS spectrum of ZCISeP QDs and the corresponding elemental maps showing the distribution of (b) Cu, (c) In, (d) Zn, (e) Se, and (f) P.

The CE process and elemental composition of synthesized QDs were verified using scanning electron microscope coupled energy-dispersive X-ray spectroscopy (SEM–EDS). The EDS elemental spectra of the QDs show characteristics of X-ray emission, corresponding to all expected elements without any detectable impurities. The quantitative SEM–EDS elemental analysis (Figure S2, inset) confirmed the atomic % of In(Zn)P QDS as 22% In, 9.9% Zn, and 17.1% P, while In(Zn)PSe QDS had 23.5% In, 10.1% Zn, 19.5% P, and 16.4% Se. The similarity in the compositions of cationic species between In(Zn)P and In(Zn)PSe QDs suggests the incorporation of Se into In(Zn)P without cation displacement. The EDS spectra of ZCISeP QDs and the corresponding elemental mapping are shown in Figure 1a. The elemental maps showed well-matched Cu, In, and Zn atoms, indicating homogeneous and even distributions of cationic species in the ZCISeP QDs. The ZCISeP QDs had an atomic composition of 8.3% Cu, 8.4% In, 8.3% Zn, 16.6% Se, and 18.5% P, indicating the formation of ZCISeP QDs with the formula CuInZn_2_Se_3_P_8_. Comparison of the initial atomic compositions of In and Zn in In(Zn)PSe QDs with those in ZCISeP QDs indicated a higher decrease in the relative composition compared with Zn^2+^, indicating the preferential replacement of In^3+^ ions by Cu^2+^ ions during the CE process. All QDs contain oxygen atoms possibly due to the carboxylate groups of the surface ligand and/or oxidation of the surface metal atom.

**Figure 2:**
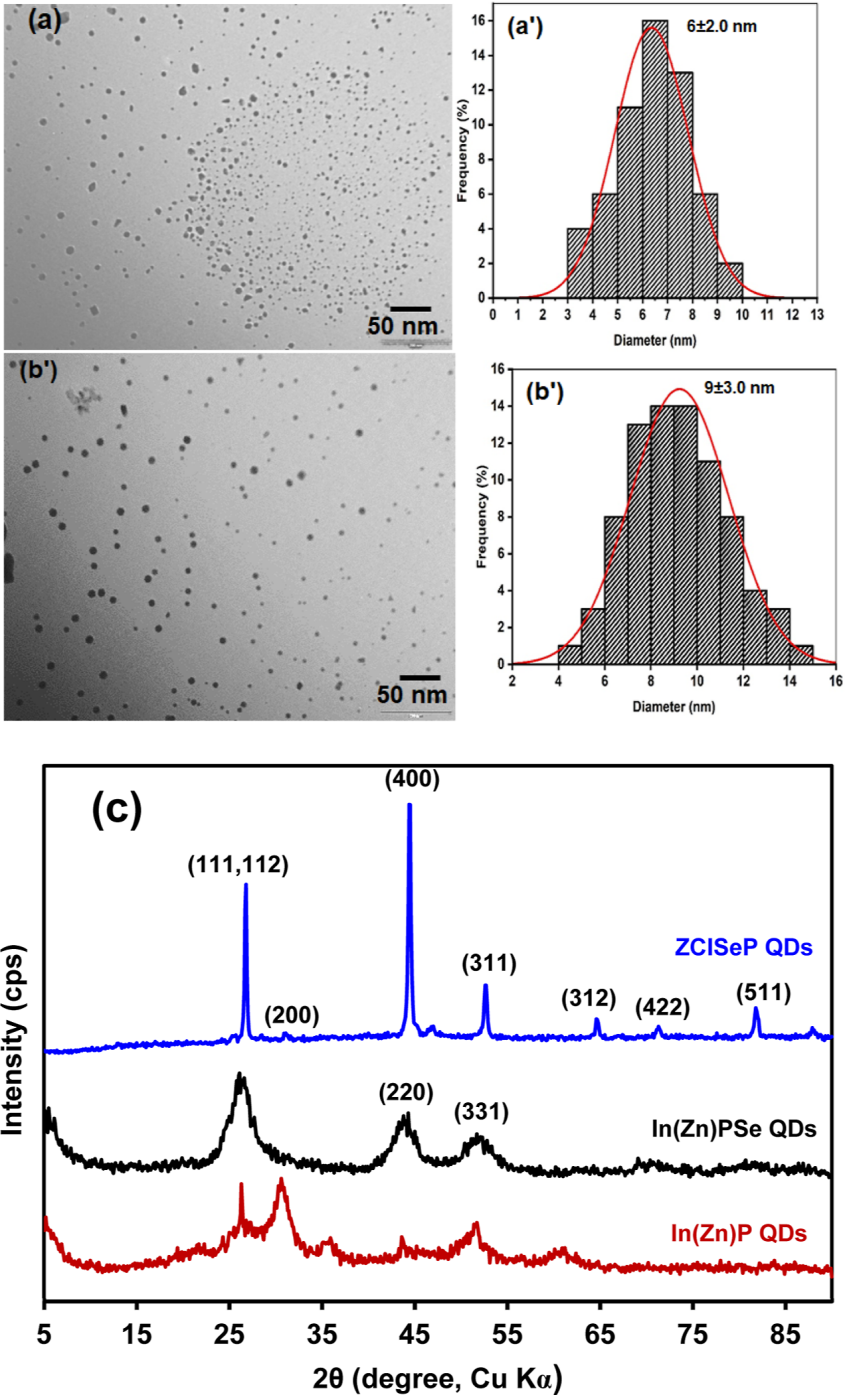
TEM images of (a) In(Zn)PSe QDs and (b) ZCISeP QDs and the corresponding size distribution histograms for (a′) In(Zn)PSe QDs and (b′) ZCISeP QDs. (c) XRD pattern of In(Zn)P, In(Zn)PSe, and ZCISeP QDs.

The transmission electron microscopy (TEM) images revealed that both the In(Zn)PSe QDs (Figure 2a) and ZCISeP QDs (Figure 2b) exhibited a quasi-spherical morphology and a uniform size distribution. It can be inferred that there are no changes in the morphology because the anionic framework remains intact during the CE process. The average particle size of the In(Zn)PSe was 6 ± 2.0 nm (Figure 2a′), and a slight increase in the particle size to 9 ± 3.0 nm (Figure 2b′) was observed for ZCISeP QDs, possibly due to the simultaneous growth process during the CE process. Powder X-ray diffractometry (XRD) was used to probe the crystalline structure and lattice parameters of the QDs. The XRD pattern of In(Zn)P QDS (Figure 2c) exhibited broad diffraction peaks at Bragg angles 26.3, 30.9, 43.5, 51.7, and 61.2°, corresponding to the (111), (200), (220), (311), and (400) crystalline planes of zinc-blende crystal structure having a *F*43*m* space group (PDF-00-032-045). The diffraction peak at 35.2° matches with the (400) crystalline plane of ZnP_2_O_7_ (PDF 04-014-330), confirming the oxidation of surface atoms.^58^ The XRD pattern of In(Zn)PSe QDs in Figure 2c showed a similar cubic zinc-blende crystalline phase, but the diffraction peaks were shifted toward higher Bragg angles at 26.6, 48.5, 51.7, and 61.2°, matching the standard diffraction pattern of In_0.75_P_0.25_Se_0.75_ (PDF-04-004-0207). There was no peak indicative of the ZnSe crystalline phase, confirming separate ZnSe QDs were not formed during the synthesis but gradient alloyed QDs structure with the possibility of an In(Zn)P-rich inner core region and a ZnSe-rich outer layer. Conversely, the XRD pattern of ZCISeP QDs exhibited sharp diffraction peaks at 26.8, 30.8, 44.7, 52.9, 64.9, 71.5, 82.1, and 88.5°, corresponding to the (111), (200), (400), (311), (312), (422), and (511) Miller indices of cubic face-centered crystalline phase of Cu_0.3_In_0.3_Zn_0.3_Se, respectively (PDF-04-020-3675). The ZCISeP QDs crystallized in the cubic crystalline plane system with a *Pm*31 space group. The analysis of the XRD pattern of synthesized ZCISeP QDs confirmed the cubic crystalline phase was maintained and no phase change occurs due to the substitutional incorporation of Cu atoms into the In(Zn)PSe crystalline lattice.

**Figure 3:**
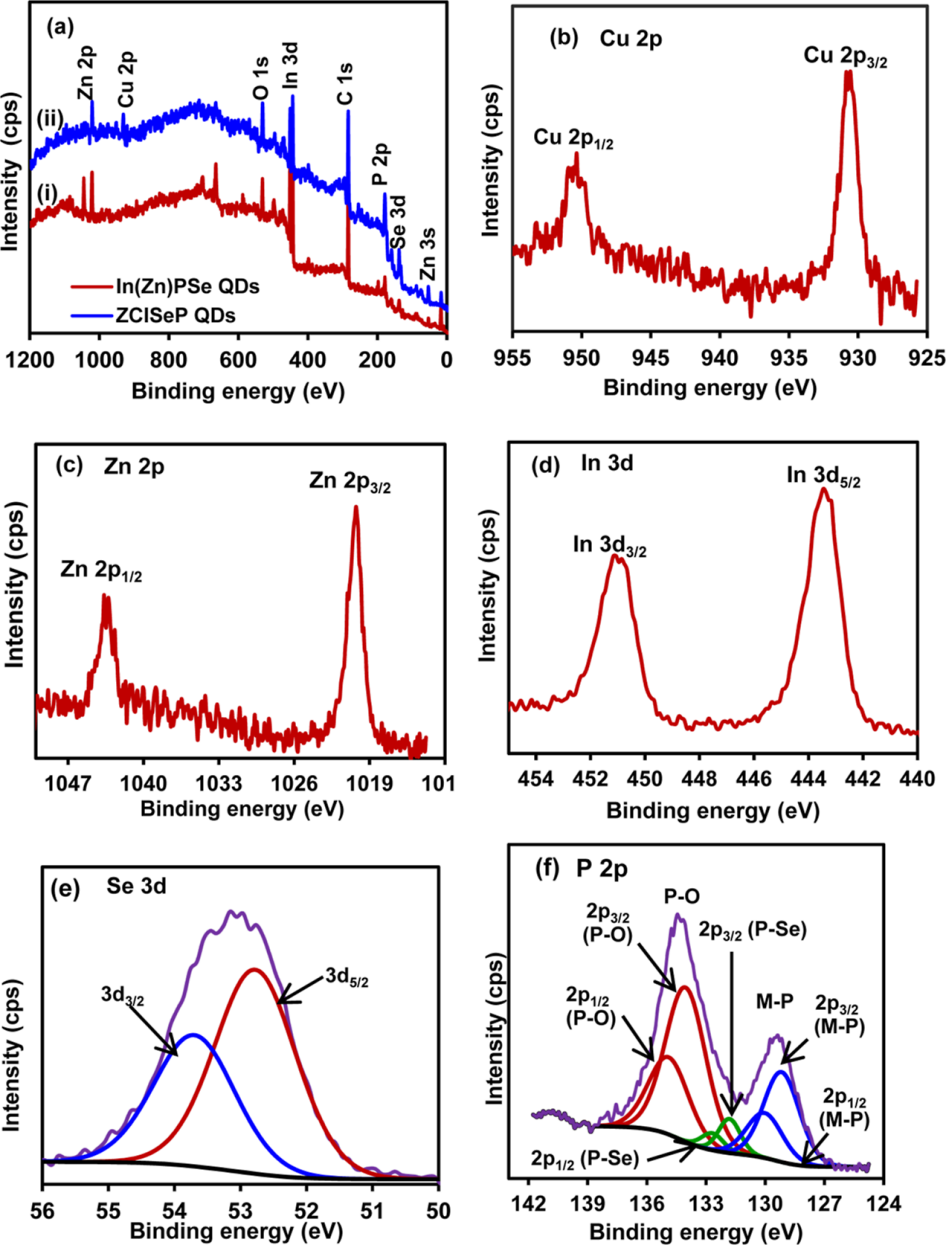
(a) XPS survey scan of (i) In(Zn)PSe and (ii) ZCISeP QDs and high-resolution XPS spectra of the (b) Cu 2p, (c) Zn 2p, (d) In 3d (e) Se 3d and (f) P 2p core-level spectra of ZCISeP QDs.

X-ray photoelectron spectroscopy (XPS) was used to analyze the elemental surface composition and chemical bonding states of the QDs. The survey spectrum of In(Zn)PSe QDs exhibited photoelectron emission corresponding to Zn 2p at 1020.5 eV, In 3d at 443.7 eV, P 2p at 138.6 eV and Se 3d at 53.7 eV (Figure 3a). Compared to In(Zn)PSe QDs, a new peak emerged at 930.7 eV in the ZCISeP spectrum and was attributed to Cu 2p. The atomic percentage (% atm) is summarized in Table S1. The ZCISeP QDs exhibited a cationic atomic surface composition of about 1:1:1 for Cu, In, and Zn, consistent with the result obtained from the EDS elemental analysis. The high-resolution Zn 2p, Cu 2p, In 3d, Se 3d, and P 2p core-level spectra of ZCISeP QDs were examined (Figure 3b–e). The core-level Cu 2p spectrum was fitted into a pair of doublets ascribed to the Cu 2p_3/2_ and Cu 2p_1/2_ energy levels with binding energy values of 931.2 and 951.0 eV, respectively (Figure 3b). The spin–orbit splitting of 19.8 eV, along with the absence of a satellite peak, confirmed the Cu^1+^ oxidation state in ZCISeP QDs. The Zn 2p spectrum was fitted into a doublet assigned to Zn 2p_1/2_ (1043.3 eV) and Zn 2p_3/2_ (1020.4 eV), with spin-split orbit coupling constant of 23 eV, indicating the chemical state of Zn atoms in the ZCISeP QDs is +2.^59^ Similarly, the In 3d spectrum in Figure 3d was fitted into 3d_3/2_ (451.1 eV) and 3d_5/2_ (443.5 eV) energy levels separated by a spin split orbit coupling of 7.6 eV, indicating the In^3+^ oxidation state. The Se 3d core-level spectra of ZCISeP QDs were fitted into a pair of doublets at 52.6 and 53.7 eV attributed to the 3d_5/2_ and 3d_3/2_, respectively (Figure 3e). Their energy splitting is approximately 0.9 eV, corresponding to the Se^2–^ chemical state of selenium bonded to metal atoms (M–Se). The high-resolution P 2p core-level spectrum was fitted into three sets of doublets with a spin split orbit coupling constant of 0.9 eV. The first pair of doublets at 129.2 and 129.9 eV was ascribed to the P 2p_3/2_ and P 2p_1/2_ of metal phosphide (M–P), confirming the phosphorus atoms bonded to the metal atoms. The second pair (132.7 and 131.8 eV) could be attributed to phosphorus bonded to selenium (P–Se). The third pair at 134.1 and 134.9 eV was assigned phosphate (P–O). XPS analysis suggests that phosphorus atoms on the surface of the QDs are oxidized.

### 3.3 Electronic Band Structure Analysis

**Figure 4:**
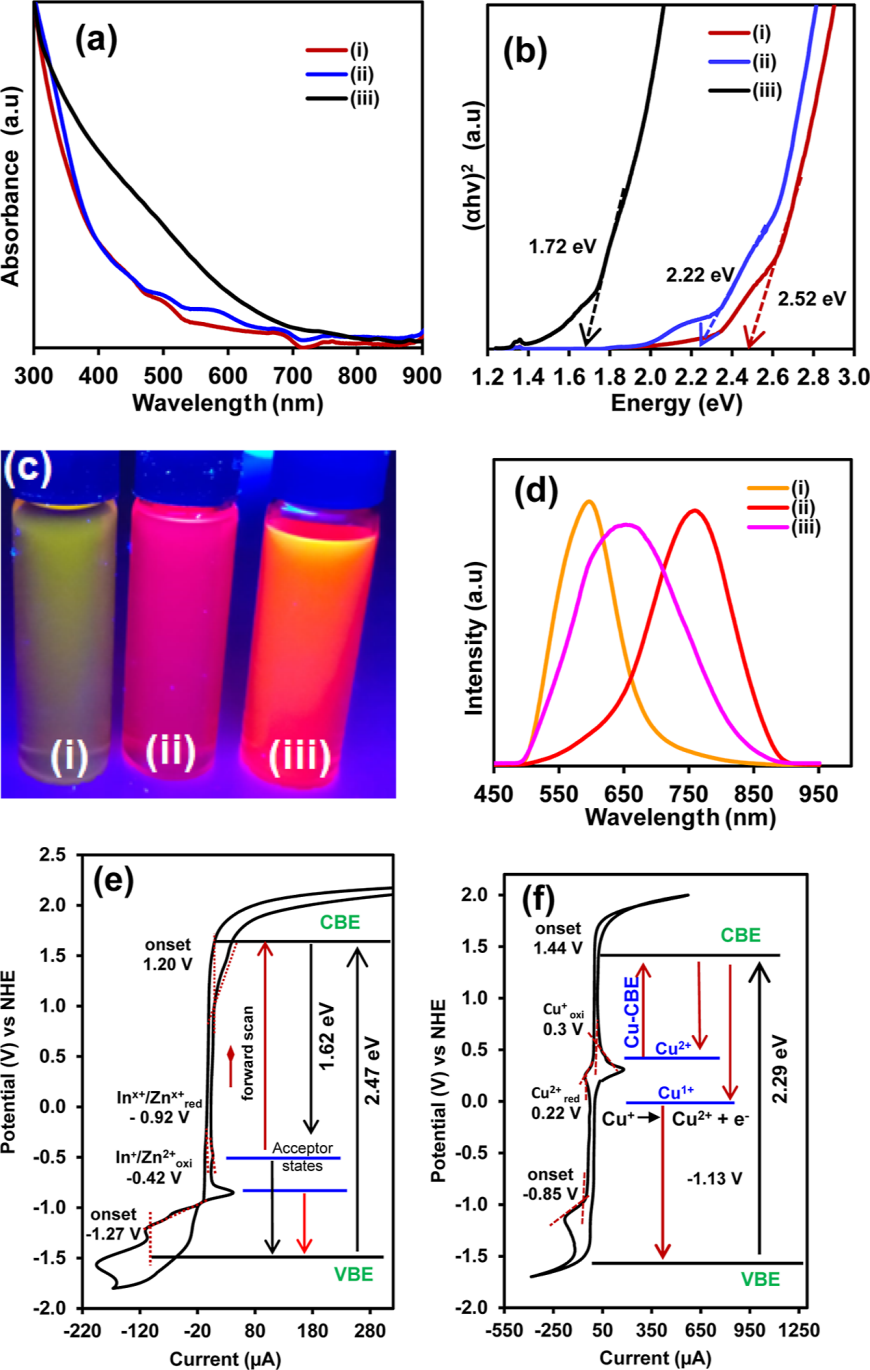
(a) UV–vis absorption spectra, (b) Tauc plot, and (c) digital photograph of (i) In(Zn)P QDs, (ii) In(Zn)PSe QDs and (iii) ZCISeP QDs and (d) corresponding fluorescence emission spectra. CV of (e) Zn(In)PSe QDs and (f) ZCISeP QDs film in PBS buffer (pH 7.4), the potential is recorded vs NHE. The inset energy diagram shows possible exciton transitions and interband gap energy states within the QDs.

Electrochemical cyclic voltammetry and steady-state optical absorption and emission spectroscopies were used to determine the electronic transition and band gap structure of the QDs (Figure 4). The UV–vis absorption spectra of both In(Zn)P and In(Zn)PSe QDs exhibited multiple peaks: a broadband edge absorption ranging from 482 and 540 nm, with maxima at 507 nm; excitonic absorption between 545 and 640 nm. In contrast, the adsorption spectra of ZCISeP QDs lacked distinct excitonic peaks within 300 and 900 nm. This featureless absorption has previously been observed in Cu-based I–III–VI multinary QDs.^60,61^ Based on the adsorption spectra, the direct optical band gap energies (*E*_g_) were extrapolated from the linear portion of the Tauc’s plot in Figure 4b. The decrease in the optical band gap energy from 2.2 eV for In(Zn)PSe QDs to 1.72 eV for ZCISeP QDs confirmed narrowing in the energy band structure due to the incorporation of Cu atoms. The In(Zn)P and In(Zn)PSe QDs emit bright yellow and red fluorescence (Figure 4c) with maximum emission wavelengths of 597 and 760 nm, respectively (Figure 4d). The quantum yield of the InP QDs was 18.4% and this increased to 76% for In(Zn)PSe QDs, indicating the ZnSe passivate surface defects and trapping state, leading to enhanced fluorescence quantum yield. Conversely, ZCISeP QDs exhibited a blue-shifted broad emission profile with a maximum wavelength of 660 nm [Figure 4d(iii)], suggesting ZCISeP QDs exhibited different fluorescence emission mechanisms compared to Zn(In)PSe due to changes in the electronic band gap structure. The fluorescence quantum yield of the ZCISeP QDs was 61%, which could be due to the introduction of Cu defect states in the QDs CV was further used to gain insight into the absolute energy of the intraband gap state of the QDs (Figure 4e,f). The procedure for the CV measurement was conducted as outlined in the apparatus and instrument sections in the Supporting Information. In principle, electrochemical oxidation corresponds to the removal of electrons from the valence band (VB) and reduction is the injection of the electron into the conduction band (CB). The onset potential of the redox process corresponds to the edges of the VB and CBE upon charge transfer (e^–^/h^+^).^62^ The CV of In(Zn)PSe QDs in Figure 4e showed two sets of cathodic and anodic peaks. The anodic peak at 1.30 V vs normal hydrogen electrode (NHE) with a corresponding onset at 1.20 V and the cathodic peak at −1.57 V with an onset at −1.27 V, correspond to an energy band gap (*E*_g_ = *E*_red_ – *E*_oxi_) value of 2.47 eV. This Eg value is in good agreement with the band-edge absorption band at 503 nm (2.45 eV) in the UV–vis spectrum of the In(Zn)PSe QDs (Figure 4a). The anodic peak at −0.52 V and the redox pair at −0.86 and −1.2 V could be attributed to In or/and Zn interstitial state or vacancies. These interstitial defects and vacancies lead to the formation of intragap energy levels, which act as donor or acceptor band states within the QDs. The band gap energy difference between the onset of the oxidation peak at −0.42 V and the CBE for the In(Zn)PSe QDs was 1.62 eV (765 nm), which corresponds to the energy value of the fluorescence emission maxima at 760 nm (1.63 eV) in Figure 4c(iii). The energy value confirmed In(Zn)PSe QDs exhibited In^+^/Zn^2+^ vacancy trap-assisted fluorescence emission process. The CV of the ZCISeP QDs in Figure 4f showed a reduction peak at −1.15 V with an onset at −0.85 V and an oxidation peak at 1.62 V with an onset potential at 1.44 V, corresponding to the VBE and CBE of the ZCISeP QDs, respectively. The electrochemical band gap of ZCISeP QDs was determined as 2.29 eV. The redox peak due to the In or/and Zn vacancies was not observed in the CV of ZCISeP QDs, confirming the substitutional incorporation of Cu^+^ atoms into the In^3+^ vacancies sites. However, on the forward scan when the potential is more oxidative, the CV of ZCISeP QDs showed anodic peaks at 0.30 and 0.43 V, ascribed to the Cu^*x*^ (*x* = +1 or +2) redox process of the Cu-vacancy and defect sites. On the reverse scan, the onset (0.39 V) of the reduction peak at 0.22 V is ascribed to the quantized VBE of the intragap Cu^x^ state. The difference in the CBE (1.44 V) and the onset of the reduction of the Cu-defect sites is 1.05 V (1196 nm) and the onset of the oxidation peak was 1.26 V (948 nm). The CV confirmed that the fluorescence emission mechanism in ZCISeP QDs is attributed to nonexcitonic Cu^*x*^ (*x* = +1 or +2) donor–acceptor-related transitions from various recombination modes via intraband energy levels, as observed in other Cu-based multinary QDs.^63−65^ These Cu^x^ intraband minigap energy states provide charge transport pathways and facilitate efficient electron tunneling processes, enabling charge carriers to tunnel easily through the QDs minibands, contributing to efficient electroconductivity of the ZCISeP QDs superlattice deposited onto the electrode.^66^

### 3.4 Electrochemical Fabrication and Characterization of Nanosensor

**Figure 5:**
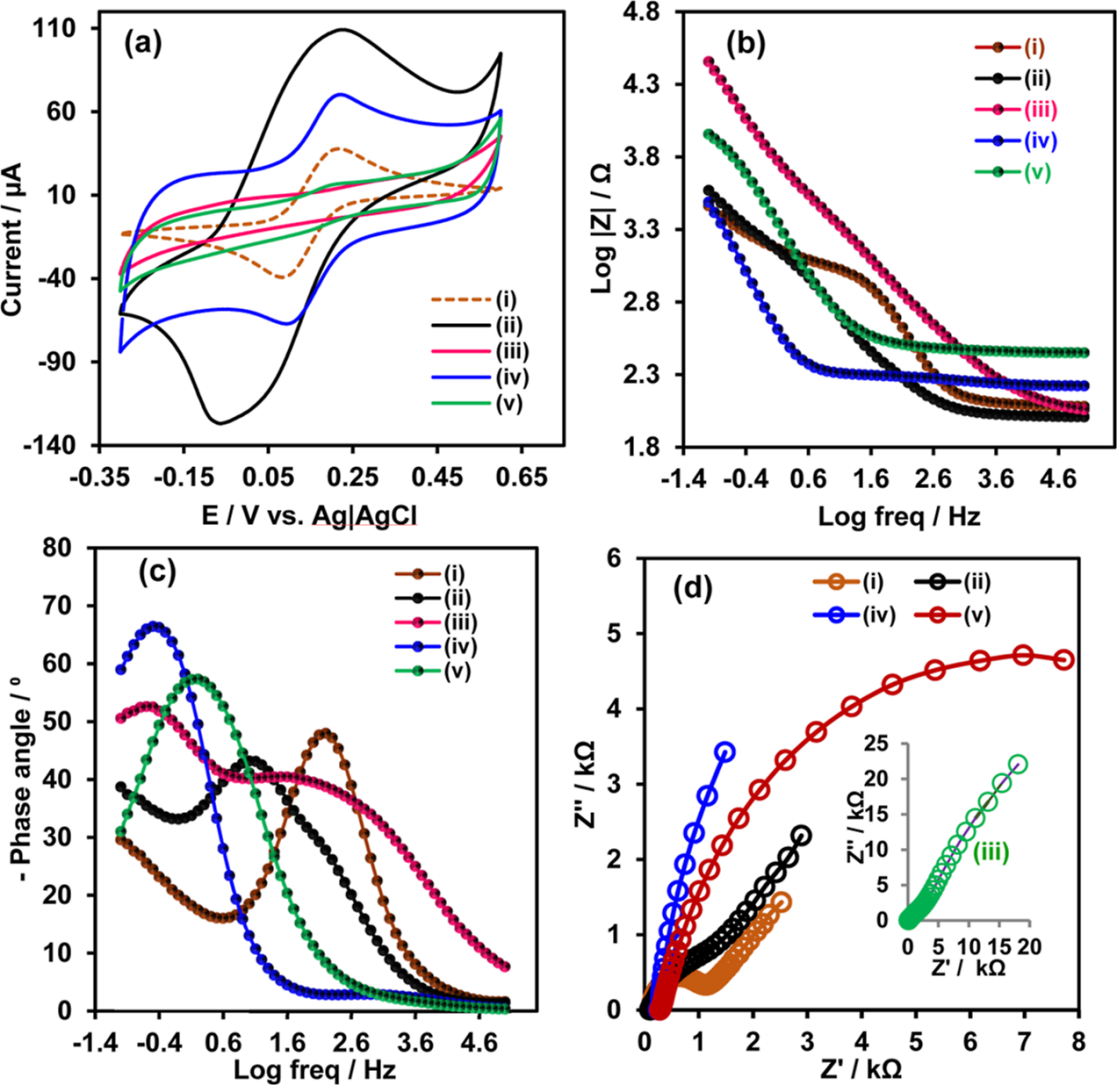
(a) CV, (b) Bode impedance plots, (c) Bode phase plots, and (d) Nyquist plot of (i) SPCE (ii) QDs/SPCE, (iii) nCoV_S1-PoPD@QDs/SPCE, (iv) MIP@QDs/SPC and (v) nCoV_S1/MIP@QDs/SPCE in PBS (pH 7.4) solution containing 2.0 mM (1:1) K_3_[Fe(CN)_6_]/K_4_[Fe(CN)_6_] and 0.1 M KCl. The inset in (d) shows the expanded region of the Nyquist plot at high frequency.

Cyclic voltammetry and electrochemical impedance spectroscopy (EIS) were used to monitor the step-by-step process involved in the fabrication of the sensor. Figure 5 shows the (a) CV, (b) Bode impedance plots, (c) Bode phase plots, and (d) Nyquist plots of (i) SPCE (ii) QDs/SPCE, (iii) nCoV2_S1-PoPD@QDs/SPCE, (iv) MIP@QDs/SPCE and (v) nCoV2_S1/MIP@QDs/SPCE in phosphate buffer solution (pH 7.4) containing 2.0 mM (1:1) of K_3_[Fe(CN)_6_]/K_4_[Fe(CN)_6_] and 0.1 M KCl. The CVs showed a reversible Fe^3+^/Fe^2+^ redox process for all of the electrodes, with each exhibiting different peak-to-peak separation (Δ*E*_p_) and current densities (Figure 5a). The CV of the SPCE [Figure 5a(i)] exhibited a Δ*E*_p_ of 110 ± 12 mV (vs Ag|AgCl), with a cathodic current (37.98 ± 0.8 μA) to anodic current (38.8 ± 1.9 μA) ratio (*I*_a_/*I*_c_) of 0.98. The modification of SPCE with ZCISeP QDs superlattice resulted in increased anodic (109.1 ± 5.4 μA) and cathodic (126.7 ± 1.3 μA) currents, indicating a 3.5-fold enhancement in electroactive surface area [Figure 5a(ii)]. Moreover, the reduction potential shifted to a lower value by 31 mV, suggesting a reduced overpotential for the [Fe(CN)_6_]^3–^ redox process at the QDs/SPCE interface. These findings confirm the enhancement of the electroactive surface area and the improvement in the electron transfer process facilitated by the ZCISeP QDs superlattice. The [Fe(CN)_6_]^3–/4–^ redox processes were completely suppressed at the nCoV2_S1-PoPD@QDs/SPCE [Figure 5(iii)] and nonimprinted PoPD@QDs/SPCE [Figure S3a(iii)], confirming an insulating film that passivates the QDs/SPCE surface was formed. The CoV2_S1-PoPD film hindered the diffusion of [Fe(CN)_6_]^3–/4–^ and impeded the electron transfer process at the electrode/electrolyte interface. Following the elution of CoV2_S1, the [Fe(CN)_6_]^3–/4–^ redox process was restored resulting in a significant increase in both anodic (70.7 ± 4.3 μA) and cathodic (66.1 ± 0.5) peak currents at the MIP@QDs/SPCE [Figure 5a(iv)]. The incubation of MIP@QDs/SPCE with nCoV2_S1 led to a decrease in the redox peak currents, confirming the binding of the nCoV2_S1 protein onto the complementary imprinted cavities. In contrast, no significant changes in the redox current were observed after incubation of nCoV2_S1 with the nonimprinted NIP@QDs/SPCE [Figure S3a(v)], confirming the absence of specific binding of the nCoV2_S1. Furthermore, EIS was utilized to evaluate the charge transport properties and capacitive behavior of the modified electrodes. The EIS data were presented in Bode impedance plots Figure 5b,c and Nyquist plot formats (Figure 5d). The EIS data were fitted with the equivalent circuits shown in Figure S3. Acceptance of the fits was contingent upon the % error value being less than 0.5%. The SPCE and QD/SPCE were well-fitted with the Randles equivalent circuit comprising a series resistor (Rs) representing the solution resistance of the electrolyte, a parallel resistor (*R*_CT_) indicating charge transfer resistance, and a constant phase element (*Q*_DL_), denoting the capacitance of the double layer at the electrode/electrolyte interface,^67^ (Figure S3b). An additional RC circuit element was incorporated in parallel to the Randles circuit elements to properly fit the EIS data for the nCoV_S1-PoPD@QDs/SPCE, MIP@QDs/SPCE, and nCoV_S_1_/MIP@QDs/SPCE (Figure S3c). This adjustment was necessary due to higher proportions of Sp2 hybridized carbon atoms in the nCoV_S1-PoPD film, which significantly influenced the kinetics of the redox processes at the modified electrodes. The additional parallel resistance (*R*_CT2_) and capacitance (*Q*_DL2_) represent the imprinted polymer charge transfer resistance and double-layer capacitance. Vertical curves observed in the frequency regions up to 4 Hz (log 0.6 Hz) of the Bode impedance plots (Figure 5b) for all of the modified electrodes indicate frequency-dependence of the impedance. The decreasing magnitude of impedance with frequency increases indicates capacitive behavior. In the high-frequency regions (≥1000 Hz) of the Bode phase plots in Figure 5c, all the electrodes showed horizontal amplitude with a phase angle tending toward 0°, indicative of uncompensated resistance primarily due to solution resistance within the electrolyte.

The maximum phase angle of the SPCE (47.98° at 158 Hz) decreased after modification with ZCISeP QDs (43.15° at 11.42 Hz), indicating a reduction in resistance in comparison to the SPCE. In the middle impedance region, the Bode impedance of QDs/SPCE, nCoV_S1-PoPD@QDs/SPCE, MIP@QDs/SPCE, and nCoV_S_1_/MIP@QDs/SPCE were linear with a slope of about 1.0 and the maximum phase angles for nCoV_S1-PoPD@QDs/SPCE (55° at 3.2 Hz), MIP@QDs/SPCE (67° at 0.40 Hz) and nCoV_S_1_/MIP@QDs/SPCE (57.3° at 1.56 Hz) tends toward −90°, indicating the nCoV_S_1_ protein and PoPD increased the double layer capacitance. The Nyquist plots of the SPCE exhibited a semicircle in the high-frequency region, indicative of an interfacial *R*_CT(1)_ value of 920 ± 33 Ω. However, a significant decrease in *R*_CT(1)_ to 106 ± 10 Ω was observed for the QDs/SPCE, confirming enhanced charge transport properties. In contrast, for the nCoV_S1-PoPD@QDs/SPCE, there was an increase in *R*_CT(1)_ to 1.48 ± 0.2 kΩ due to the presence of the insulating nCoV_S1-PoPD film. Furthermore, an additional *R*_CT(2)_ of 26.5 ± 4 kΩ associated with the nCoV_S1-PoPD film was noted. However, both *R*_CT(1)_ and *R*_CT(2)_ decreased to 44.8 ± 9 Ω and 5.31 ± 0.1 kΩ, respectively, for the MIP@QDs/SPCE. Following the incubation of the QDs/SPCE with nCoV_S1, the value of *R*_CT(1)_ and *R*_CT(2)_ increased to 504 ± 20 Ω and 8.91 ± 0.7 kΩ, respectively, indicating the rebinding of nCoV_S1 onto the MIP@QDs/SPCE induced resistance to the [Fe(CN)_6_]^3–/4–^ redox process.

### 3.5 Surface Morphology and Composition of Nanosensor Interfaces

**Figure 6:**
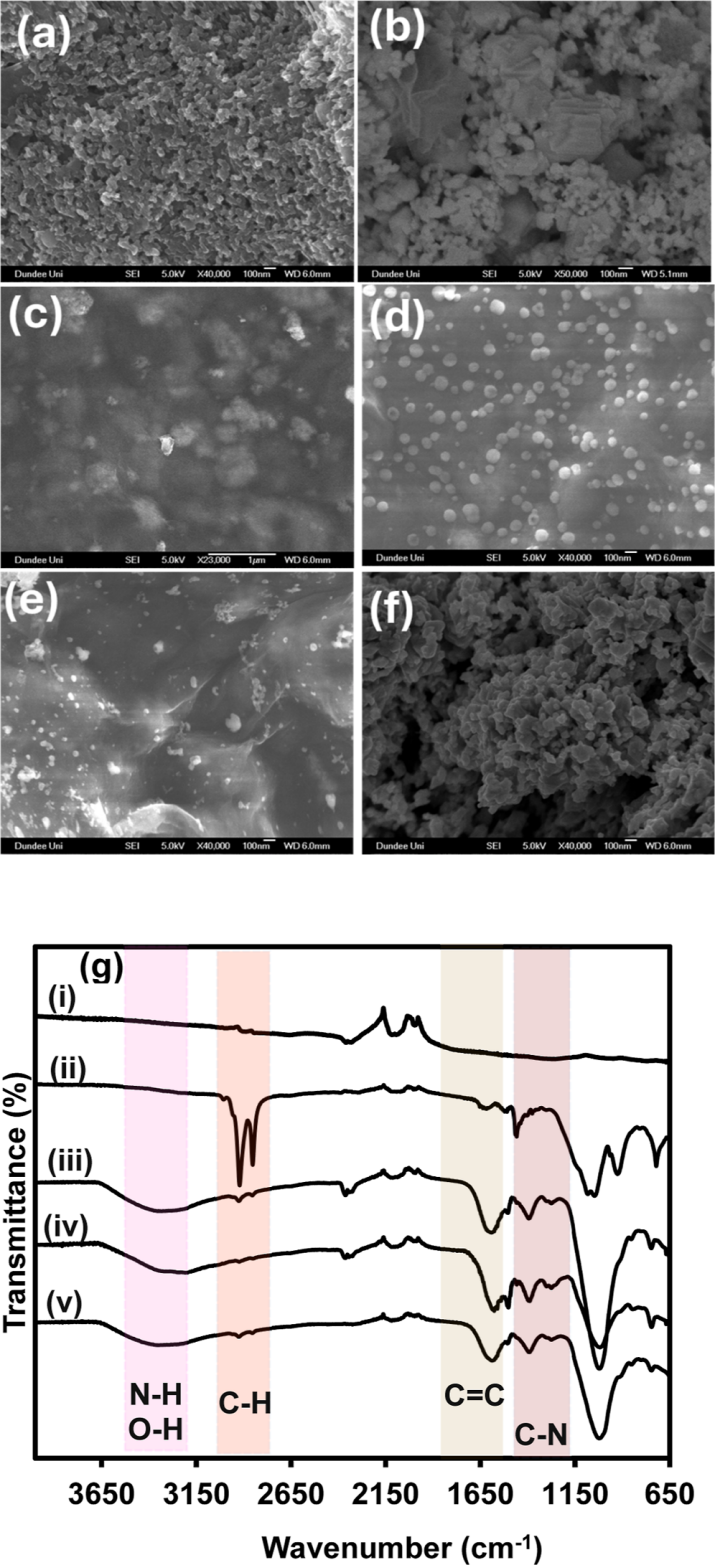
SEM images of (a) SPCE, (b) QDs/SPCE, (c) PoPD@QDs/SPCE, (d) nCoV_S1-PoPD@QDs/SPCE, (e) NIP@QDs/SPCE, (f) MIP@QDs/SPCE modified electrode surfaces, and (g) FTIR spectra of (i) bare SPCE, (ii) QDs/SPCE, (iii) PoPD@QDs/SPCE, (iv) nCoV_S1-PoPD@QDs/SPCE, and (v) MIP@QDs/SPCE modified electrode surfaces.

The SEM images of the working electrode area of the various modified electrodes reveal the surface structural properties of their interfaces. Figure 6 shows the high-resolution SEM images of the (a) SPCE, (b) QDs/SPCE, (c) PoPD@QDs/SPCE, (d) nCoV_S1-PoPD@QDs/SPCE, (e) NIP@QDs/SPCE, and (f) MIP@QDs/SPCE obtained at a magnification of 40,000 using an in-lens detector. The high-resolution SEM image of the bare SPCE (Figure 6a) revealed a highly textured and rough surface composed of highly interconnected ultrafine carbon particles. Upon the deposition of ZCIPSe QDs, the SEM image of QDs/SPCE (Figure 6b) clearly shows a densely packed agglomeration of the QDs forming a clustered superlattice structure on the SPCE. This close packing of QDs indicates successful dot-to-dot coupling necessary for efficient electron transfer within the QDs superlattice and is crucial for improved sensor performance. The SEM images of the nanosensor fabricated via electropolymerization of oPD monomer onto the QDs/SPCE, but without (PoPD@QDs/SPCE, Figure 6c) and with the nCoV_S1 template (nCoV_S1-PoPD@QDs/SPCE, Figure 6d), showed a smoother surface film compared to that of the QDs/SPCE, with embedded particles visible within the film. Notably, the SEM image of the nonimprinted PoPD@QDs/SPCE surface (Figure 6c) appeared smother with less monodispersed particles observed within the film compared to the nCoV_S1-PoPD@QDs/SPCE surface (Figure 6d). This suggests that the presence of the nCoV_S1 template results in a less dense polymer film, which is advantageous for target recognition and binding. The SEM image of MIP@QDs/SPCE (Figure 6f) obtained after treatment with the elution solution shows a distinctive interconnected layer with microporous morphology, confirming the successful removal of the nCoV_S1 template. The formation of these imprinted cavities is critical as they provide specific binding sites for biorecognition of SARS-CoV2-virus, enhancing the selectivity and sensitivity of the nanosensor.

The FTIR spectra were acquired from the fabricated electrode surface to investigate the surface functional groups. As shown in in Figure 6g(i), the FTIR spectra of the bare SPCE surface did not exhibit any noticeable absorption bands within the typical mid-infrared region (4000 to 650 cm^–1^). However, after modifying the SPCE surface with QDs, the FTIR spectrum of the QDs/SPCE surface in Figure 6g(ii) showed distinct absorption peaks due to the aliphatic C–H stretching vibrations at 2922 and 2853 cm^–1^, and bending vibrations at 1459 cm^–1^, attributed to the aliphatic C–H groups of the MPA ligands on the QDs surface. The peaks at 723 and 1049 cm^–1^ are associated with the C–S and C–O stretching vibrations of MPA, confirming the presence of MPA at the surface of the QDs/SPCE superlattice interface. After the electropolymerization process, the intensity of the characteristic vibrational bands due to the QDs decreased, indicating that the QDs were embedded within the polymer film for the PoPD@QDs/SPCE [Figure 6g(iii)] and nCoV_S1-PoPD@QDs/SPCE [Figure 6g(iv)] surfaces. New characteristic bands at 1502 and 1587 cm^–1^, corresponding to the benzenoid and quinoid ring C=C stretching vibrations of the PoPD polymer backbone, were observed in the FTIR spectra of PoPD@QDs/SPCE and nCoV_S1-PoPD@QDs/SPCE, indicating successful polymerization and formation of the PoPD film. The FTIR spectra of PoPD@QDs/SPCE and nCoV_S1-PoPD@QDs/SPCE were similar. The broad absorption band observed between 3540 and 3084 cm^–1^ can be attributed to the overlap of the N–H and aromatic C–H asymmetric stretching vibrations of the PoPD and possibly the amino acid side groups of nCoV-2. This broad peak also indicated the presence of hydrogen-bonded O–H stretching vibrations of entrapped water molecules within the polymer film. The 1390 cm^–1^ peak indicates C–N stretching vibrations of the polymer quinonoid ring, while the band at 1276 cm^–1^ is attributed to the C–N stretching vibrations of the aliphatic side chains. The peak at 980 cm^–1^ is assigned to C–H out-of-plane bending vibrations in the aromatic ring, while the peaks at 842 and 745 cm^–1^ correspond to the out-of-plane C–H bending vibrations. After treatment with the eluent to remove the n-CoV-2 template, the FTIR spectra of MIP@QDs/SPCE did not show significant changes, indicating that the polymer layer was stable and did not degrade during the elution process.

### 3.6 Optimization of Nanosensor Fabrication and Detection Condition

**Figure 7:**
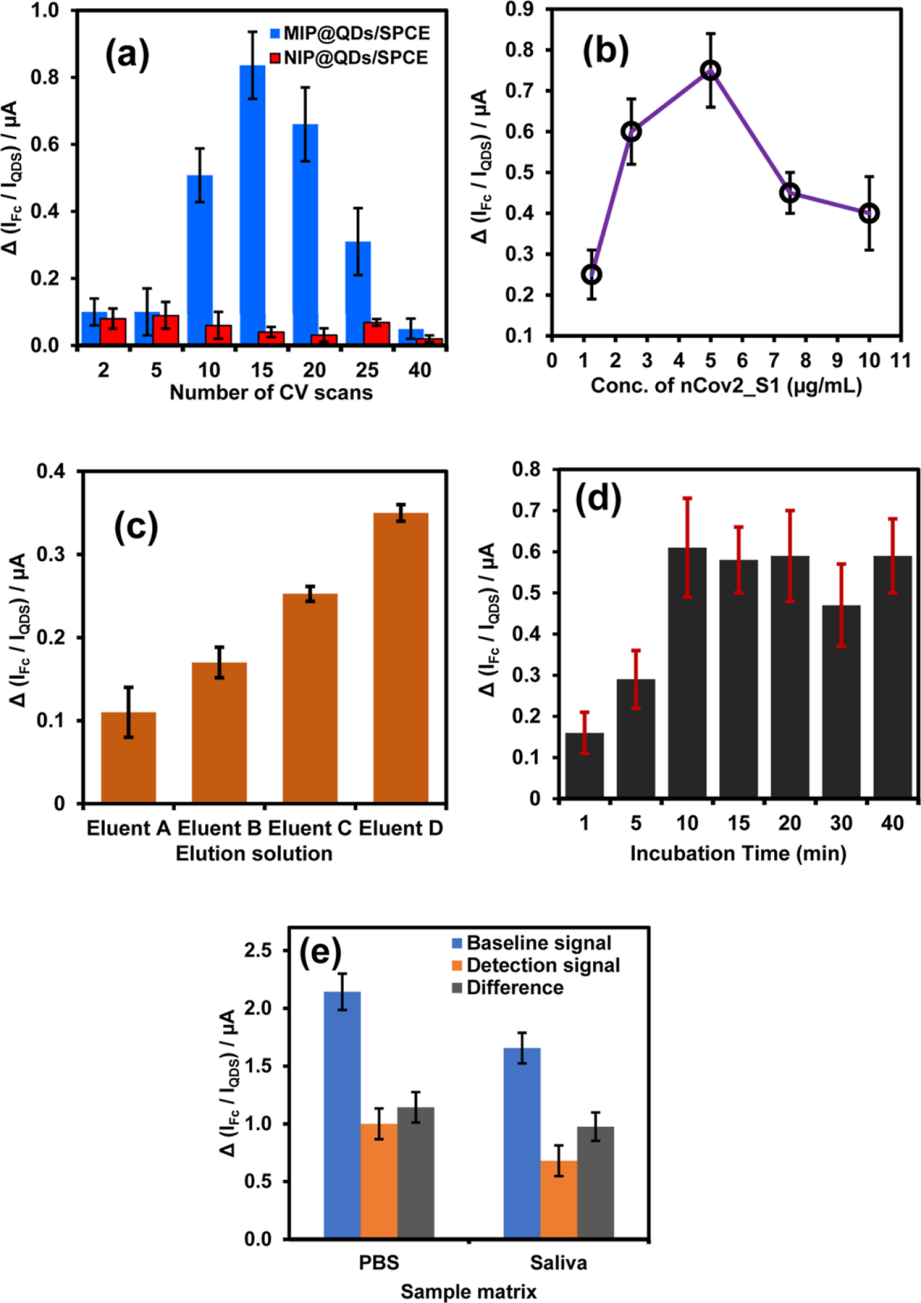
Optimization of sensor performance and fabrication steps (a) effect of numbers of scans applied for the electropolymerization of nCoV2_S1/oPD monomer onto the QDS/SPCE, (b) concentration of nCoV2_S1 template in the monomer, (c) type of eluent solution used, (d) incubation time for the removal of nCoV2_S1 template, and (e) effect of sample matrix on the sensitivity of MIP@QDs/SPCE sensor toward the detection of 100 pg/mL of nCoV2_S1. All data were obtained from the average of the DPV measurement from three electrodes prepared independently. The error bars represent the standard deviation of three independent measurements.

Various parameters related to the sensor fabrication and assay procedures were optimized to enhance the sensitivity and analytical performance of the MIP@QDs/SPCE sensor for detecting the nCoV2_S1 protein. The optimal parameters were determined by analyzing changes in the DPV peak current response to 50 pg mL^–1^ of nCoV2_S1. The number of cyclic voltammetry scans employed during the electropolymerization of PoPD-nCoV2_S1 onto the QDs/SPCE determined the thickness of the imprinted PoPD-nCoV2_S1 layer (Figure 7a). The thickness significantly influences the analytical performance of the nanosensor. As depicted in Figure 7a, the sensor fabricated with fewer scans (2–5 scans) exhibited low sensitivity. There was no noticeable difference between the MIP@QDs/SPCE and the NIP@QDs/SPCE sensor responses to 50 pg mL^–1^ of nCoV2_S1 protein. This suggests that a thin MIP film, formed with 2–5 scans, was unable to effectively confine the nCoV2_S1 and there were no imprinted cavities for the recognition of the target. In contrast, the MIP@QDs/SPCE sensor fabricated with higher numbers of scans (10–25 scans) exhibited significantly higher signal intensity, with optimum sensitivity observed at 15 CV scans. Increased scans led to a thick MIP film that effectively imprinted numerous template molecules, resulting in higher sensitivity due to increased numbers of complementary cavities for nCoV2_S1 binding. Additionally, the thicker MIP film maintained mechanical stability and imprinted cavities structure during the template removal and assay process. However, sensors fabricated with higher voltammetric scans (>15 scans) showed decreased sensitivity. This decline in sensitivity could be attributed to the formation of a dense, multilayered thicker film, which hinders efficient template removal and rebinding of nCoV2_S1. The NIP@QDs/SPCE sensor fabricated with higher numbers of scans (10–40 scans) exhibited a decrease in the current response compared to lower scans (<15 scans), suggesting that the thicker film also suppresses nonspecific binding of nCoV2_S1. Therefore, 15 scans were the optimum number of cycles for fabricating the MIP@QDs/SPCE. The effect of nCoV2_S1 template concentration ranging from 1 to 12.5 μg/mL on the sensor sensitivity was investigated while maintaining a fixed concentration of oPD (10 mM). Figure 7b shows that the signal intensity increases with increasing nCoV2_S1 concentration up to 5 μg/mL and decreases afterward. This trend suggests that the number of recognition cavities within the MIP film increases with higher protein concentrations. However, the decrease observed at a concentration exceeding 5 μg/mL could be attributed to the inefficient formation of binding cavities due to the crowding effect at higher concentrations. The sensitivity of the sensor is also influenced by the efficiency of the elution buffer in removing the nCoV2_S1 template to form stable imprinted cavities. The MIP@QDs/SPCE sensors were fabricated using different elution solutions, including eluent A (1:1 v/v mixture of ethanol and 0.25 mM acetic acid), eluent B (1:1 v/v ethanol and 0.25 mM NaOH), eluent C (1:1 v/v ethanol and 135 mM NaCl, 1% SDS and 0.1% Tween 20), and eluent D (1:1 v/v ethanol, 0.25 mM NaOH, 135 mM NaCl, 1% SDS, 0.1% Tween 20), with an elution time of 60 min. As shown in Figure 7c, the sensor fabricated by eluting with eluent D exhibits the highest sensitivity, indicating the efficient removal of the template without changes in the structural properties of the MIP layer. The assay time was optimized by incubating the MIP@QDs/SPCE with nCoV2_S1 for varying time intervals (1 to 40 min), Figure 7d. The sensor response increased with longer incubation times, reaching the highest response at 10 min and levels up beyond 10 min indicating that all available binding sites were occupied within 10 min. Therefore, the optimal incubation time for detection of nCoV2_S1 was 10 min. For the practical application of the sensor for detection, the effect of the raw saliva matrix on the sensor signal was evaluated. The baseline signal of MIP@QDs/SPCE in the raw saliva and PBS was compared with the detection signal after incubation with 100 pg/mL of nCoV2_S1 spiked saliva and PBS samples. As shown in Figure 7e, the baseline signal intensities after incubation with raw saliva were lower than the signal intensities for the PBS. However, the difference in the detected signal of the sensor after incubation with nCoV2_S1 spiked saliva (0.99 μA) and PBS (1.14 μA) was 0.15 μA, indicating the binding of nCoV2_S1 to MIP@QDs/SPCE was not compromised by the saliva matrix and constituent. Hence the sensor has comparable sensitivity in PBS and saliva.

### 3.7 Signal Transduction Mechanism of MIP@QDs/SPCE and Ratiometric Quantitative Detection of nCoV2_S1

**Figure 8:**
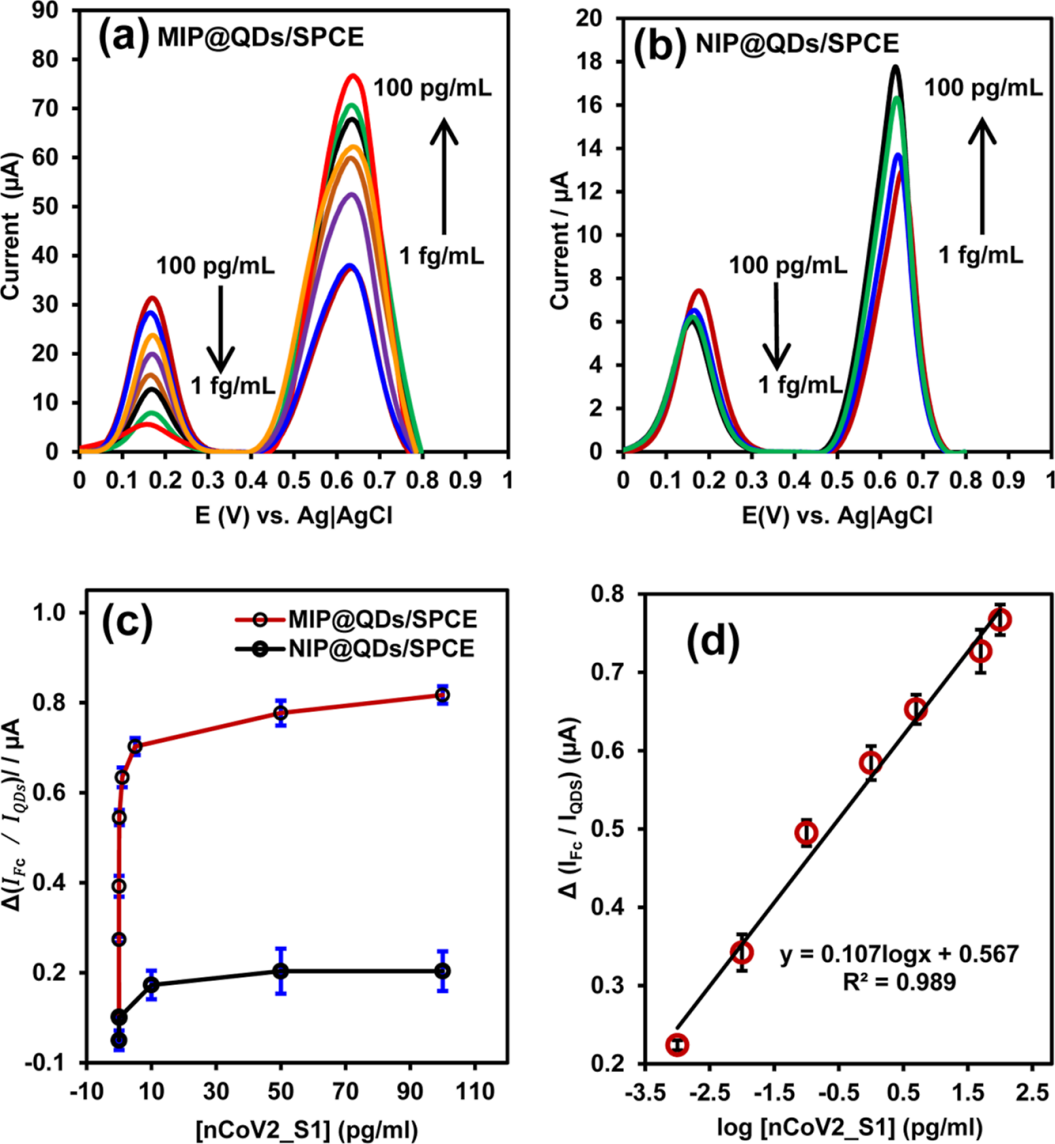
DPV plot of (a) MIP@QDs/SPCE and (b) NIP@QDs/SPCE in 0.1 M PBS buffer (pH 7.4) containing 2.0 mM (1:1) K_3_[Fe(CN)_6_]/K_4_[Fe(CN)_6_] and 0.1 M KCl, after incubation with human saliva containing different concentrations of nCoV2_S1 (0.001–100 pg/mL). (c) The dose–response curve showing changes in the ratio of (*I*_Fc_/*I*_QDs_) peak current intensity against the concentrations of nCoV2_S1 and (d) linear calibration plot of *I*_Fc_/*I*_QDs_ against the logarithm of nCoV2_S1 concentrations in human saliva. The error bars represent the standard deviation of three independent measurements on different fabricated nanosensors.

Using the optimized assay conditions, the quantitative detection of nCoV2_S1 in human saliva samples was demonstrated with MIP@QDs/SPCE and the validation of the sensor performance was compared to NIP@QDs/SPCE. The nanosensor signal was quantified through DPV measurements in [Fe(CN)_6_]^3–/4–^ redox mediator before and after incubation with raw saliva samples spiked with varying concentrations of nCoV2_S1. As shown in Figure 8a, the DPV showed two anodic peaks at 0.17 and 0.64 V vs Ag|AgCl. To elucidate the mechanism underlying the role of the QDs in generating the dual voltammetric signal, an MIP@SPCE sensor without the QDs superlattice was fabricated for comparison. As shown in Figure S4, the DPV response of the MIP@SPCE in [Fe(CN)_6_]^3–/4–^ showed only an anodic peak at 0.17 V, attributed to the oxidation of Fe^2+^ to Fe^3+^. The anodic peak at 0.64 V was not observed at the MIP@SPCE, indicating that this peak is due to the charging effect of the QDs superlattice.^22^ At the MIP@QDs/SPCE (Figure 8a), the anodic peak current for [Fe(CN)_6_]^3–/4–^ (*I*_Fc_) decreased, while the peak current of the QDs (*I*_QDs_) increased with increasing concentrations of nCoV2_S1 from 0.001 to 100 pg/mL. This suggests that the binding of nCoV2_S1 onto the complementary MIP cavities induced a gating effect, hindering the diffusion of [Fe(CN)_6_]^3–/4–^ to the underlying QDs/SPCE interface.^68^ Consequently, fewer [Fe(CN)_6_]^3–/4–^ ions migrated to the QDs/SPCE, resulting in a decreased anodic peak current at 0.17 V as the concentrations of nCoV2_S1 increased. Additionally, the binding of the nCoV2_S1 to the MIP@QDs/SPCE induces a charge redistribution at the QDs superlattice interface.^22,60^ This charge redistribution enhanced the quantum mechanical coupling between the QDs particles, promoting the charge transfer process at the interface,^22,60^ and resulting in higher current at 0.64 V with increasing nCoV2_S1 concentrations. Conversely, the anodic peak currents of [Fe(CN)_6_]^3–/4–^ and QDs did not change significantly as the concentrations of nCoV2_S1 increased for the NIP@QDs/SPCE nanosensor (Figure 8b). This indicates that the MIP@QDs/SPCE nanosensor response was specific to the binding of nCoV2_S1 onto the cavities.

To ensure excellent sensing reliability, a ratiometric analytical signal based on the Δ(*I*_Fc_/*I*_QDs_) estimate by eq 1 was used for the quantitative detection of nCoV2_S1.
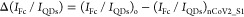
1where  and  are the ratio of the peak current intensities before (without nCoV2_S1) and after incubation with nCoV2_S1, respectively. The dose–response curves of Δ(*I*_Fc_/*I*_QDs_) against the concentrations of nCoV2_S1 exhibited saturated binding isotherms that were well fitted to the Langmuir–Freundlich model Figure 8c. The affinitive binding constant (*K*_D_) which is the nCoV2_S1 concentration required to provide half of the maxima response was calculated as 0.54 ± 0.2 pg/mL of nCoV2_S1. The maximum binding capacity (*B*_max_), extrapolated from the dose–response curve as the concentrations of the saturation signal, was estimated as 50.0 pg/mL for the MIP@QDs/SPCE sensor. The capability of the MIP@QDs/SPCE to recognize the nCoV2_S1 protein was estimated from the imprinting factor (IF) using eq 2, where *B*_max(MIP@QDs)_ and *B*_max(NIP@QDs)_ are the maxima binding capacities of imprinted and nonimprinted sensors, respectively. The IF was calculated as 5.3, indicating efficient binding of nCoV2_S1 to the MIP@QDs/SPCE.
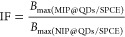
2

The plot of relative intensities Δ(*I*_Fc_/*I*_QDs_) against the logarithm of the concentrations of nCoV2_S1 demonstrates a linear correlation in the range of 0.001 to 100 pg mL^–1^ (1.13–1309 fM). The linear regression equation was given by Δ(*I*_Fc_/*I*_QDs_) = 0.107 log[nCoV2_S1] (pg/mL) + 0.57, *R*^2^ = 0.989. It is worth noting that despite recent criticism regarding the use of semilogarithmic (semilog) plots in analyzing analytical sensing data,^69^ it remains suitable for linear fittings of binding isotherms involving biomolecular recognition and electroanalytical data processing due to the underlying principles of these methods.^70^ The limit of detection (LoD) and other analytical parameters using a semilog fit remain valid.^70^ The LoD and limit of quantification (LoQ) were determined from the slope (s) of the semilog regression equation and the standard deviation (σ) of the *I*_Fc_/*I*_QDs_ for the baseline signal (without nCov2_S1), using 3σ/slope and 10σ/slope, respectively. The (σ) of the baseline signal was calculated as 0.012 (*n* = 6). The LoD and LoQ of the MIP@QDs/SPCE were found to be 0.34 pg/mL (4.53 fM) and 1.12 pg/mL (14.9 fM), respectively, based on the semilog plot. This indicated that MIP@QDs/SPCE can accurately detect nCoV2_S1 proteins in the femtomolar range, indicating high sensitivity. Considering that there are approximately 24 to 40 spike proteins on each SARS-CoV-2,^19^ with each spike protein containing an S1 subunit, the LoD of the MIP@QDs/SPCE was estimated to fall between 1.09 × 10^5^ and 6.82 × 10^4^ copies/μL of SARS-CoV-2 virions using eq 3. Given that the SARS-CoV2 viral load reaches up to 6.6 × 10^5^ copies/μL after 4 days of infection.^71−73^ the MIP@QDs/SPCE showed high potential for detection of SARS-CoV-19 in human saliva samples during the early stage of infection.
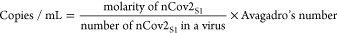
3

The analytical performance of the MIP@QDs/SPCE nanosensor was compared to other electrochemical sensors reported for the detection of the SARS-CoV-2 virus in the literature. The comparison is summarized in Table S2. The LoD of the MIP@QDs/SPCE sensor (4.5 fM) is comparable to that of antibody-based detection (14 fM),^74^ Angiotensin-converting enzymes-2 receptor (6.17 fM),^75^ and other MIP-based recognition element sensors.^16,19^ However, the developed MIP@QDs/SPCE had a wide linear dynamic range compared to those of most of the other reported sensors, providing an advantage of saliva analysis without dilution. The wide linear range could be attributed to the high surface area and surface-to-volume ratio of the QDs and the semilog logarithm data fittings. Also, the ratiometric signal provided a built-in correction factor, which helps to improve the accuracy of the sensor.

### 3.8 Selectivity and Reproducibility of MIP@QDs/SPCE Nanosensor

**Figure 9:**
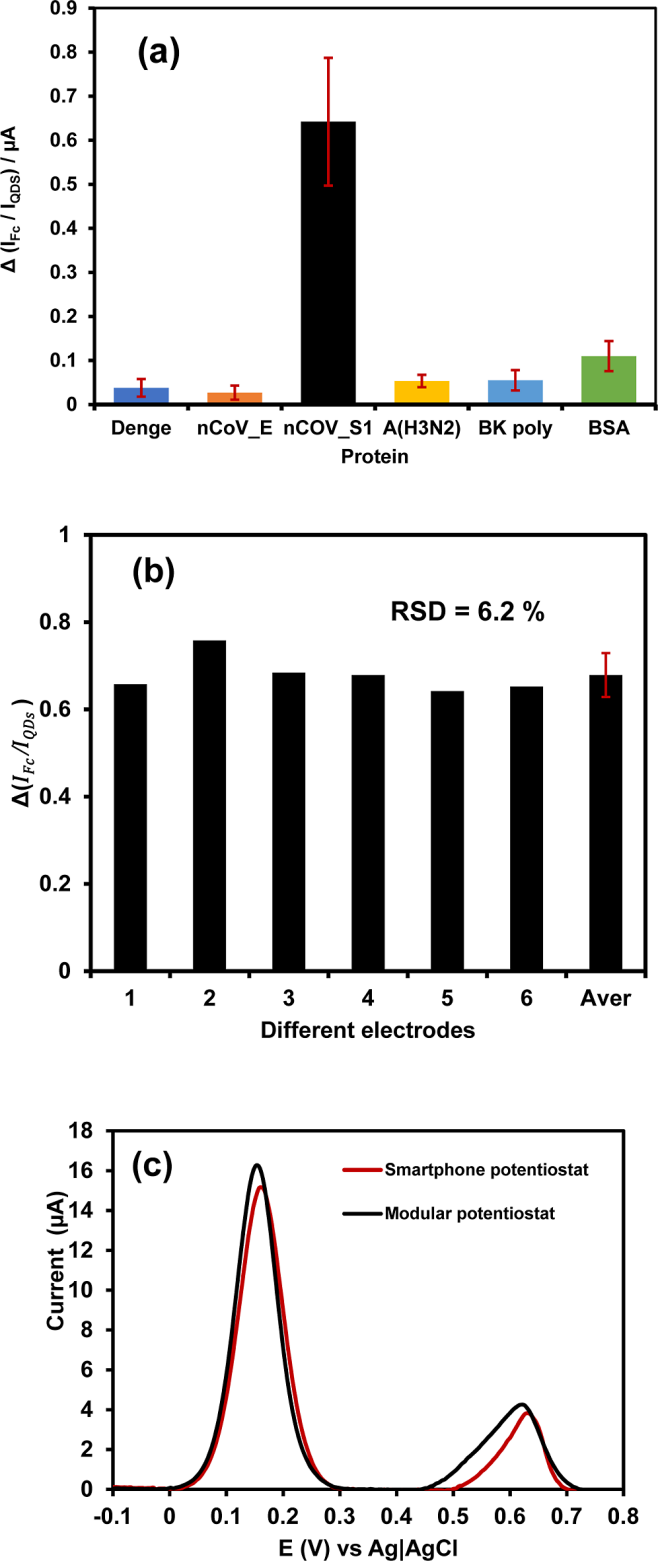
(a) Selectivity of MIP@QDs/SPCE sensor showing its response to 100 pg/mL of different proteins (Dengue virus protein, nCoV_E, nCoV_S1, influenza A(H3N2), BK polymer virus, and BSA), (b) ratiometric current responses of six independently prepared electrodes to 100 pg/mL of nCoV2-S1 spiked saliva samples, showing sensor-to-sensor reproducibility, and (c) comparison of the DPV of smartphone potentiostat to the modular electrochemical potentiostat recorded with the same MIP@QDs/SPCE nanosensor. The error bars are the standard deviation of triplicate measurement from the same electrode after a regeneration step.

The selectivity of the MIP@QDs/SPCE was evaluated by comparing the assay response for nCoV2-S1 to other viral and nonviral proteins at the same concentrations (100 pg/mL) in spiked human saliva samples. The rationale for the selection of the protein was based on the potential to coexist in human saliva samples, the molecular weight, and the isoelectric point. Figure 9 shows a comparison of the nanosensor current signal response to nCoV2-S1 and other proteins. The error bar in Figure 9a represents the standard deviation of triplicate measurements (*n* = 3) on the same electrode following a regeneration step and rebinding of the analyte. The nanosensor showed a significantly higher response to nCoV2-S1 compared with the other interferent proteins. The sensor could discriminate between nCoV2-S1 (75 kDa) compared with bovine serum albumin (BSA, 66 kDa), and Influenza A H3N2 protein (AH3N2, 80 kDa) with similar molecular weight. The sensor response to nCoV2-S1 was about 20 times greater than its response to the SARS-CoV2 envelope protein (ncov_E) and H3N2 protein, which could potentially coexist with nCoV2-S1 protein in saliva. These confirmed the high selectivity of our developed sensor for nCoV2-S1. The high standard deviation observed for the nCOV_S1 protein in Figure 9a could be attributed to the variations in the nanosensor response after each regeneration step, suggesting that the MIPs@QDs/SPCE is most suitable for single-use measurements. To evaluate the sensor-to-sensor reproducibility, six MIPs@QDs/SPCE nanosensors were independently fabricated under the same conditions to detect 100 pg/mL nCoV2-S1 spiked saliva samples. The DPV responses from these nanosensors, shown in Figure S5, displayed similar current response magnitudes. The background signal-corrected responses (Figure 9b) had a relative standard deviation of 6.2%, indicating good reproducibility between the nanosensors.

### 3.9 Integration of MIP@QDs/SPCE with Smartphone-Based Potentiostat for POC Diagnostics

To improve the portability and meet the ASSURED criteria for point-of-care applicability, we investigated the integration of the MIP@QDs/SPCE with a commercially available smartphone-based potentiostat (Sensit smart) for quantitative analysis of ncov_S1 in saliva. Our system consists of a Samsung smartphone equipped with the Android app PStouch Analytical V2.8 software. To verify whether the detection current results of the smartphone device are as reliable as those of the modular electrochemical workstation, we compared the DPV current response of the same MIP@QDs/SPCE on the two potentiostats using the same settings. As shown in Figure 8b, the potential and current intensities of the differential voltammogram from the two potentiostats are comparable. Therefore, the smartphone potentiostat can replace the electrochemical workstation for the detection of nCoV2_S1.

## 4 Conclusions

In summary, we have demonstrated the synthesis of a novel multinary Zn–Cu–In–Se–P (ZCISeP) QD via a CE reaction, and we have also fabricated a ZCISeP QDs superlattice film-modified screen-printed electrode, which served as a transducer for the development of a new ratiometric electrochemical nanosensor. Electron microscopy and spectroscopic and electrochemical techniques were used to characterize the QDs. The ZCISeP QDs showed a monodispersed particle morphology from the TEM analysis while also exhibiting a cubic-face centered crystal structure from XRD analysis. From the electrochemical analysis, the ZCISeP QDs superlattice showed a copper-vacancy-related redox process and excellent charge transfer properties. The QDs-based superlattice was functionalized with an MIP possessing highly specific cavities for the nCoV2_S1 protein binding via a surface imprinting electrochemical polymerization process. Leveraging the high surface area and efficient electron transfer properties of the QDs superlattice, coupled with the specificity of MIP cavities and the ratiometric signal mode, our nanosensor achieved enhanced analytical performance compared to previously reported sensing systems. The electrochemical nanosensor had a detection limit in the femtomolar range, a wide detection range, and selectivity for the SARS-CoV-2 S1 protein in saliva samples. The ratiometric signal provided inherent self-nonspecific signal correction, ensuring the reliability of analytical data. Moreover, the integration of the MIP@QDs/SPCE with a smartphone-based potentiostat enabled the utilization of our nanosensor for screening in outpatient departments and home testing for viral infections. This can be readily adapted for telemedicine applications during pandemics. The introduction of the use of a ZCISeP QDs superlattice as a material for electrochemical sensor development expands the application scope of QDs within the biomedical field. Notably, this study opens an avenue for developing QDs-based electrochemical sensing systems targeting various disease biomarkers, expanding the toolbox available for disease diagnostics in the future.

## Supporting Information Available

Instrumentation and method of analysis; preparation of copper-oleate; SEM–EDS elemental spectra, FTIR spectra, CV for electropolymerization synthesis of protein imprinted polymer electrode; EIS equivalent circuit elements; and XPS elemental composition analysis and comparison of the fabricated MIP@QDS/SPCE sensor to other recently reported electrochemical sensors for SARS-CoV-2 detection (PDF)
