# Supplementary material for: Molecularly Imprinted Viral Protein Integrated Zn–Cu–In–Se–P Quantum Dots Superlattice for Quantitative Ratiometric Electrochemical Detection of SARS-CoV-2 Spike Protein in Saliva
Source: ACS Appl Nano Mater. 2024 Jul 24;7(15):17630–47. doi: 10.1021/acsanm.4c02882 (PMC11320384; doi:10.1021/acsanm.4c02882)
Supplement: Supplementary file 1 — an4c02882_si_001.pdf [file an4c02882_si_001.pdf]

Supporting Information

Molecularly Imprinted Viral Protein Integrated Zn-Cu-In-Se-P Quantum Dots Superlattice for Quantitative Ratiometric Electrochemical Detection of SARS-COV-2 Spike Protein in Saliva

Kayode Omotayo Adeniyi, Kayode Oyinlola, Ojodomo J. Achadu, Herve Menard, Federico Grillo, Zhugen Yang, Oluwasesan Adegoke*

[a] *Leverhulme Research Centre for Forensic Science, School of Science & Engineering, University of Dundee, Dundee, DD1 4GH, UK*

[b] *School of Health and Life Sciences, and National Horizon Centre, Teesside University, Middlesbrough TS1 3BA, UK.*

[c] *School of Water, Energy and Environment, Cranfield University, Cranfield, MK43 0AL, UK.*

[d] *School of Chemistry, University of St Andrews, St Andrews KY16 9ST, UK.*

* Correspondence: o.adegoke@dundee.ac.uk; KAdeniyi001@dundee.ac.uk.

Leverhulme Research Centre for Forensic Science, School of Science & Engineering, University of Dundee, Dundee, DD1 4GH, UK

1. Apparatus and instruments

Electrochemical measurements were carried out using AUTOLAB PGSTAT204 Potentiostat/Galvanostat equipped with Nova 2.1.6 software and FRA32 M for impedance measurements and analysis. Smartphone-based electrochemical measurements were carried out using PalmSense Sensit Smart potentiostat equipped with an EmStat Pico module and controlled via the PStouch Android software. A three-electrode setup using screen-printed Ag|AgCl pseudo-reference and carbon working and counter electrodes was used for all measurements. The difference between the potential measurement using the AutoLab and Sensit Smart is less than ±0.005 A. The CV measurement for analysis of the electronic band gap structure of the QDs was recorded in phosphate buffer (pH 7.4) within a potential of -1.5 to 1.5 V vs (Ag|AgCl) starting from 0.0 V for the forward scan. The potential values were converted to the normal hydrogen potential (NHE) for band gap evaluation. UV-vis absorption and fluorescence emission spectra were acquired using a Varian Cary Eclipse spectrophotometer. Transmission electron microscopy (TEM) images were acquired using JEOL JEM-1200EX operated at 80 kV. X-ray diffraction (XRD) analysis was carried out using a Siemens D5000 diffractometer with Cu Kα radiation (λ = 1.54056 nm) and data were obtained in the range of 5-90° using a step size of 0.1° 2θ step, 3 sec count time per step and 0.066° slit width. Field emission scanning electron microscopy images (FSEM) were acquired from JEOL JSM 7400 F integrated with an Oxford Instruments Inca EDX spectrometer for Energy dispersive X-ray (EDX) analysis operated at 15 KV. Raman analysis was carried out using an in-house built microprobe system equipped with a continuous wave laser source emitting at 633 nm, the Oriel MS257 monochromator fitted with the Andor Newton EMCCD detector, TE cooled to -70°C. The backscattering configuration was used for the signal collection. The incident power was 6.8 mW and spectra were recorded using a 40x objective (Plan Fluor, Nikon), at a 2.5 s accumulation time with a total of 2 accumulations, a slit width of 150 mm and a 1200 lines/mm grating. XPS was carried out on a Kratos Axis Ultra-DLD photoelectron

spectrometer equipped with an Al monochromatic X-ray source, and the data were analysed using CasaXPS software. The spectra were calibrated based on the C 1*s* peak from adventitious carbon. Quantification was performed based on the area of peaks of interest (i.e. Zn $2p_{3/2}$, Cu $2p_{3/2}$, In $3d_{5/2,3/2}$, P $2p_{3/2,1/2}$ and Se $3d_{5/2,3/2}$) after background subtraction.

2. Preparation of Cu-oleate

$CuCl_2.2H_2O$ (1.71 g, 10 mmol) and sodium oleate (9.13 g, 30 mmol) were dissolved in a solvent mixture comprising ethanol (40.0 mL), hexane (70.0 mL) and MilliQ H_2O (30 mL). This mixture was then heated at 80 °C and allowed to react for 4 hr. After completion of the reaction, the organic layer was separated using a separating funnel and washed three times with Milli-Q water (100 mL). The resulting green-colored copper-oleate was dissolved in chloroform (50 mL) and purified through washing and centrifugation using ethanol-chloroform (100 ml, 3:1 v/v) and ethanol-acetone (100 ml, 1:1 v/v) mixtures. The product was dried at dried at 50 °C, yielding pure Cu-oleate in the form of a green powder. The stock Cu-oleate precursor solution (1.2 mmol/L) was prepared by dissolving Cu-oleate (15 mg) in octylamine (20 mL) by sonication on a water bath at 50 °C until dissolution was complete.

3. Supporting Figures

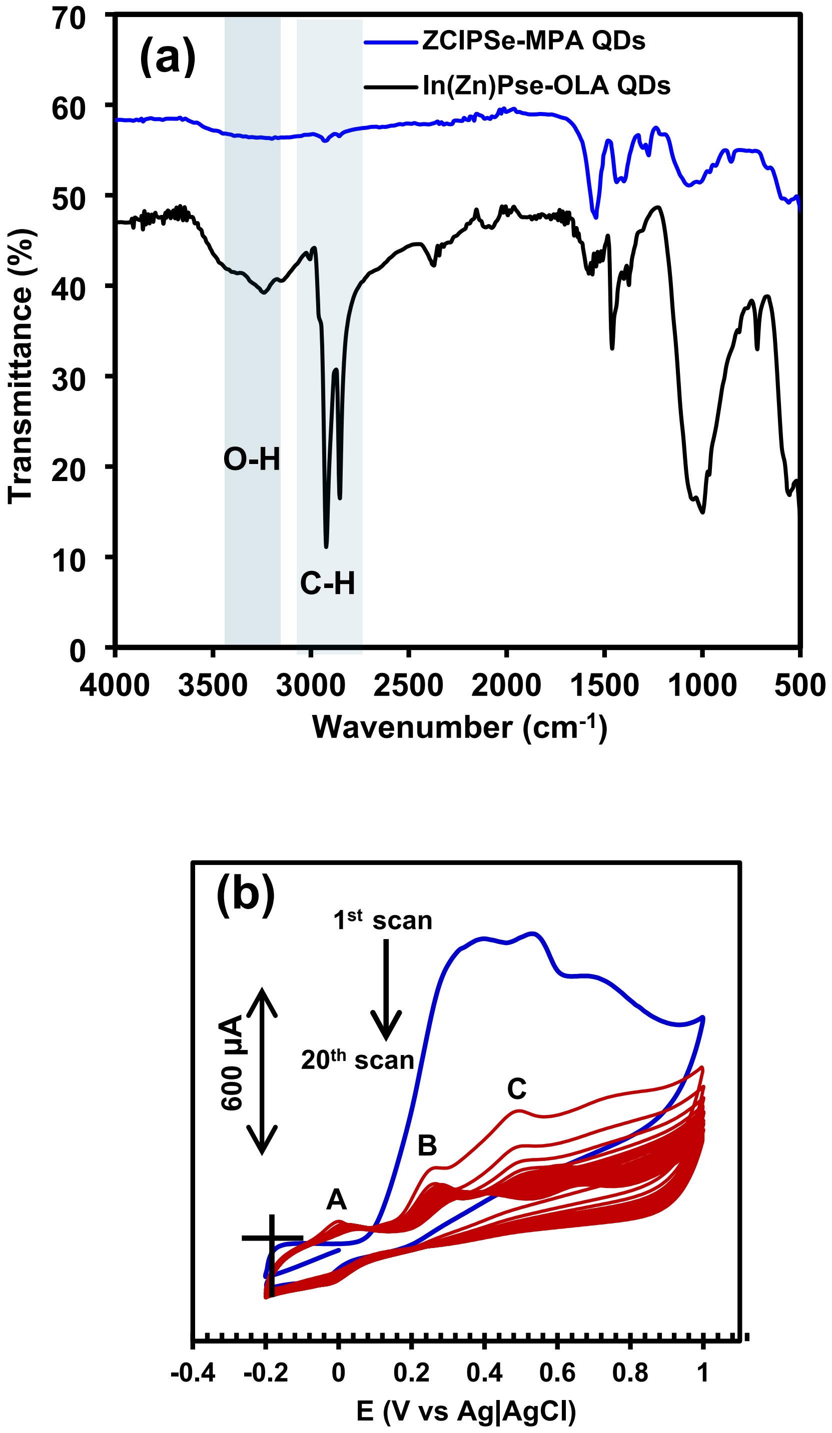

Fig. S1. (a) FTIR spectra of ZCIPSe QDs before and after ligand exchange with MPA confirming the successful ligand exchange process and (b) CV of QD/SPCE in 10 mM oPD monomer solution containing 2.5 µg/ml of nCoV2_S1 at a scan rate of 50 mV/s.

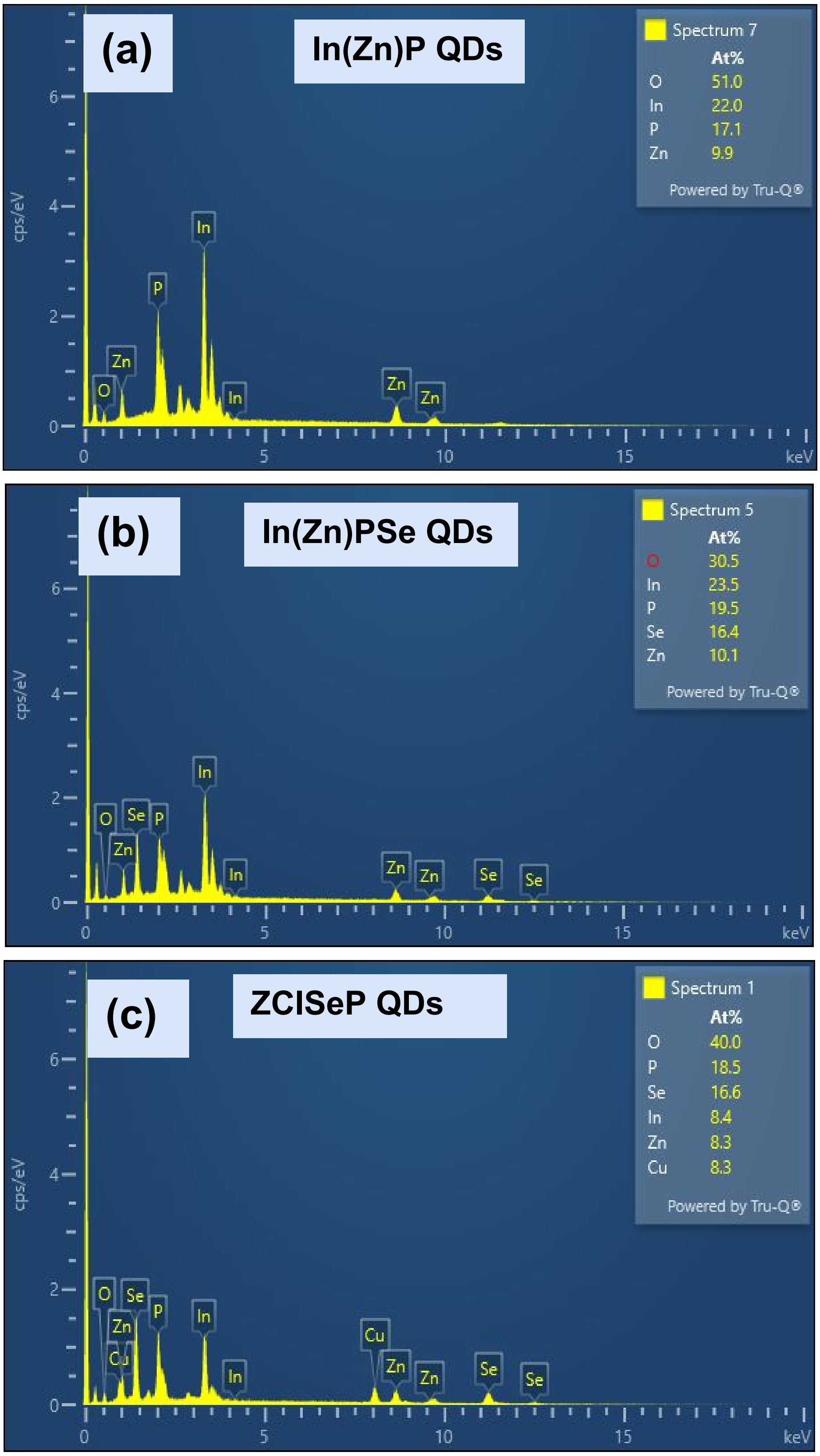

Fig. S2. EDS spectra of (a) In(Zn)P, (b) In(Zn)PSe, and (c) ZCIPSe QDs, with an inset showing the atomic elemental composition.

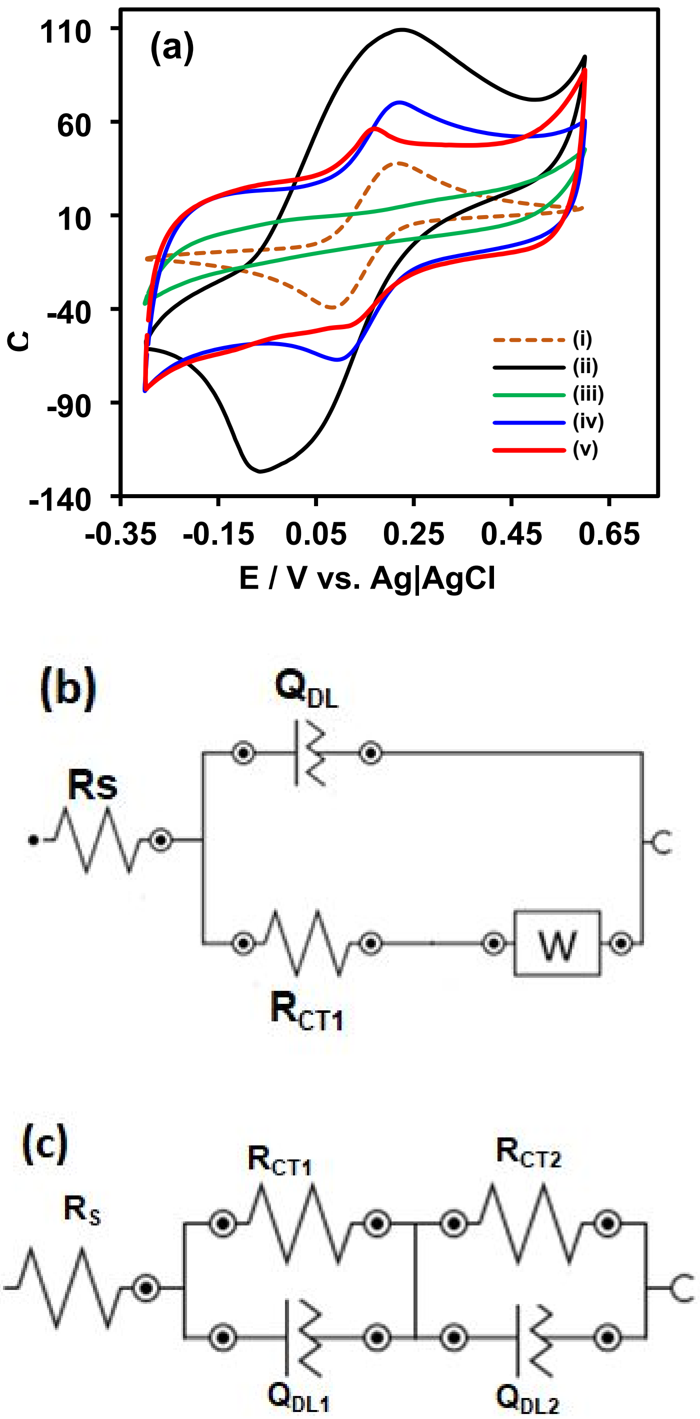

Fig. S3. (a) CV of (i) SPCE (ii) QDs/SPCE, (iii) nCoV_S1-PoPD@QDs/SPCE, (iv) MIP@QDs/SPC, and (v) nCoV_S1/NIP@QDs/SPCE in PBS (pH 7.4) solution containing 2.0 mM (1:1) $K_3[Fe(CN)_6]$: $K_4[Fe(CN)_6]$ and 0.1 M KCl. Equivalent circuit for fitting the EIS data of (b) bare

SPCE and QDs/SPCE and (b) nCoV_S1-PoPD@QDs/SPCE, MIP@QDs/SPCE and nCoV_S_1/MIP@QDs /SPCE.

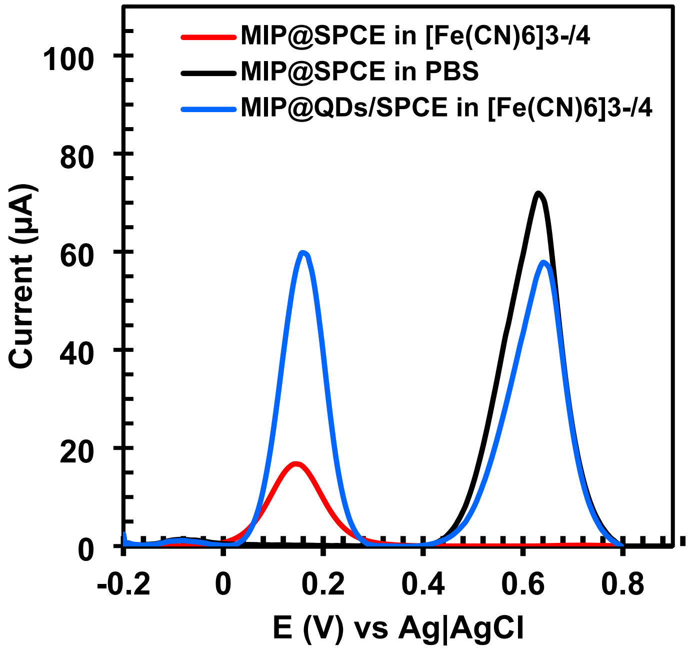

Fig. S4. DPV of MIP@SPCE in PBS buffer (PH 7.4), MIP@SPCE and MIP@QDs/SPCE in PBS (pH 7.4) solution containing 2.0 mM (1:1) $K_3[Fe(CN)_6]$: $K_4[Fe(CN)_6]$ and 0.1 M KCl.

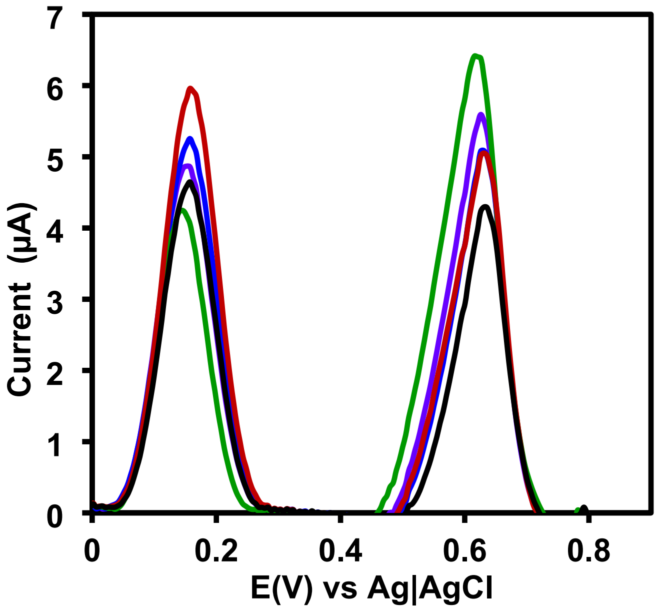

Fig. S5. DPV response of independently prepared MIP@QDs/SPCE nanosensor to 100 pg/mL of nCoV_S1.

Table S1 % Atomic elemental composition of the QD obtained from XPS analysis.

QDs	Elemental composition (% atm)						
	Zn 2p (1020.5 eV)	Cu 2p (930.7 eV)	In 3d (443.7 eV)	Se 3d (53.7 eV)	P 2p (137.6 eV)	C 1s (284.9 eV)	O 1s (530.7 eV)
In(Zn)PSe	3.13	-	6.01	1.61	9.36	71.55	8.34
ZCISeP	1.51	1.34	1.16	3.3	21.45	58.96	12.25

Table S2. Comparison of different electrochemical sensors for SARS-COV-2 detection

**Transducer interface**	**Linear range**	**LoD pg/ml**	**Ref**
Au/CSH/SARS-CoV-2 Ab/BSA/analyte/Ab2-Fc	-	0.08	1
FTO/AuNPs/SARS-CoV-2 Ab	-	120 fM	2
SPE/CNF/N-protein/Ab	0.1-1000 pg/ml	0.8	3
Framework MIL-53(Al)-Aptamer	0.025-50 pg/ml	8.33	4
AuE/CoFeBDCCNH$_2$-CoFe$_2$O$_4$ MOF/SARS-CoV-2 Ab	10 -100 fg/ml	14 fM	5
MIP/MP-Au-SPE	2 - 40 pg/ml	0.7	6
Au-TFME/ncovNP-MIP	2-111 fM	0.7 (15 fM)	7
AuE-ACE-BSA-Nafion (Rapid 1.0)	0.00133 -1.33 fM	6.17 pg/ml	8
Au-TFME/ncov2_S1-MIP	0-200 fM	4.8 (64 fM)	9
MIP@ZCISeP QDs/SPCE	1.13-1390 fM	0.34 (4.5 fM)	This study

SPE: screen printed electrode, Au: gold, MP: Microporous, MIP: Molecularly imprinted polymer, ACE: Angiotensin-converting enzymes-2, Fc: ferrocene, Ab: capture antibody, Ab2: detection antibody, BSA: Bovine serum albumin, CNF: carbon nanofiber.

References

(1) Kowalczyk, A.; Kasprzak, A.; Ruzycka-ayoush, M.; Podsiad, E.; Demkow, U.; Grudzinski, I. P.; Nowicka, A. M. Sensors and Actuators : B . Chemical Ultrasensitive Voltammetric Detection of SARS-CoV-2 in Clinical Samples. *Sensors Actuators B Chem.* **2022**, *371* (132539). https://doi.org/10.1016/j.snb.2022.132539.

(2) Roberts, A.; Mahari, S.; Shahdeo, D.; Gandhi, S. Label-Free Detection of SARS-CoV-2 Spike S1 Antigen Triggered by Electroactive Gold Nanoparticles on Antibody Coated Fluorine-Doped Tin Oxide (FTO) Electrode. *Anal. Chim. Acta* **2021**, *1188*, 339207. https://doi.org/10.1016/j.aca.2021.339207.

(3) Eissa, S.; Zourob, M. Development of a Low-Cost Cotton-Tipped Electrochemical Immunosensor for the Detection of SARS-CoV - 2. *Anal. Chem.* **2021**, *93*, 1826–1833. https://doi.org/10.1021/acs.analchem.0c04719.

(4) Tian, J.; Sun, D.; Chen, Z. Electrochimica Acta An Electrochemical Dual-Aptamer Biosensor Based on Metal-Organic Frameworks MIL-53 Decorated with Au @ Pt Nanoparticles and Enzymes for Detection of COVID-19 Nucleocapsid Protein. **2021**, *387*. https://doi.org/10.1016/j.electacta.2021.138553.

(5) Palanisamy, S.; Lee, L.; Kao, C.; Chen, W.; Wang, H.; Shen, S.; Jian, J.; Yuan, S. F.; Kung, Y.; Wang, Y. Sensors and Actuators : B . Chemical One-Step-One-Pot Hydrothermally Derived Metal-Organic-Framework-Nanohybrids for Integrated Point-of-Care Diagnostics of SARS-CoV-2 Viral Antigen / Pseudovirus Utilizing Electrochemical Biosensor Chip. *Sensors Actuators B. Chem.* **2023**, *390* (April), 133960. https://doi.org/10.1016/j.snb.2023.133960.

(6) Amouzadeh Tabrizi, M.; Fernández-Blázquez, J. P.; Medina, D. M.; Acedo, P. An Ultrasensitive Molecularly Imprinted Polymer-Based Electrochemical Sensor for the Determination of SARS-CoV-2-RBD by Using Macroporous Gold Screen-Printed Electrode. *Biosens. Bioelectron.* **2022**, *196* (October 2021). https://doi.org/10.1016/j.bios.2021.113729.

(7) Raziq, A.; Kidakova, A.; Boroznjak, R.; Reut, J.; Öpik, A.; Syritski, V. Development of a Portable MIP-Based Electrochemical Sensor for Detection of SARS-CoV-2 Antigen. *Biosens. Bioelectron.* **2021**, *178* (November 2020). https://doi.org/10.1016/j.bios.2021.113029.

(8) Torres, M. D. T.; de Araujo, W. R.; de Lima, L. F.; Ferreira, A. L.; de la Fuente-Nunez, C. Low-Cost Biosensor for Rapid Detection of SARS-CoV-2 at the Point of Care. *Matter* **2021**, *4* (7), 2403–2416. https://doi.org/10.1016/j.matt.2021.05.003.

(9) Ayankojo, A. G.; Boroznjak, R.; Reut, J.; Opik, A. Sensors and Actuators : B . Chemical Molecularly Imprinted Polymer Based Electrochemical Sensor for Quantitative Detection of SARS-CoV-2 Spike Protein. **2022**, *353*.
